# Identification of a transporter Slr0982 involved in ethanol tolerance in cyanobacterium *Synechocystis* sp. PCC 6803

**DOI:** 10.3389/fmicb.2015.00487

**Published:** 2015-05-18

**Authors:** Yanan Zhang, Xiangfeng Niu, Mengliang Shi, Guangsheng Pei, Xiaoqing Zhang, Lei Chen, Weiwen Zhang

**Affiliations:** ^1^Laboratory of Synthetic Microbiology, School of Chemical Engineering and Technology, Tianjin UniversityTianjin, China; ^2^Key Laboratory of Systems Bioengineering (Ministry of Education), Tianjin UniversityTianjin, China; ^3^SynBio Research Platform, Collaborative Innovation Center of Chemical Science and Engineering (Tianjin)Tianjin, China

**Keywords:** ethanol, tolerance, transporter, metabolomics, *Synechocystis*

## Abstract

Cyanobacteria have been engineered to produce ethanol through recent synthetic biology efforts. However, one major challenge to the cyanobacterial systems for high-efficiency ethanol production is their low tolerance to the ethanol toxicity. With a major goal to identify novel transporters involved in ethanol tolerance, we constructed gene knockout mutants for 58 transporter-encoding genes of *Synechocystis* sp. PCC 6803 and screened their tolerance change under ethanol stress. The efforts allowed discovery of a mutant of *slr0982* gene encoding an ATP-binding cassette transporter which grew poorly in BG11 medium supplemented with 1.5% (*v/v*) ethanol when compared with the wild type, and the growth loss could be recovered by complementing *slr0982* in the Δ*slr0982* mutant, suggesting that *slr0982* is involved in ethanol tolerance in *Synechocystis*. To decipher the tolerance mechanism involved, a comparative metabolomic and network-based analysis of the wild type and the ethanol-sensitive Δ*slr0982* mutant was performed. The analysis allowed the identification of four metabolic modules related to *slr0982* deletion in the Δ*slr0982* mutant, among which metabolites like sucrose and *L*-pyroglutamic acid which might be involved in ethanol tolerance, were found important for *slr0982* deletion in the Δ*slr0982* mutant. This study reports on the first transporter related to ethanol tolerance in *Synechocystis*, which could be a useful target for further tolerance engineering. In addition, metabolomic and network analysis provides important findings for better understanding of the tolerance mechanism to ethanol stress in *Synechocystis*.

## Introduction

Bioethanol production through microbiological processes has drawn greater attention in recent years due to increasing cost of non-renewable fossil fuels and environmental concerns related to over-utilization of fossil fuels (Hahn-Hagerdal et al., 2006). Various native or engineered microorganisms, such as *Saccharomyces cerevisiae* (Eiadpum et al., 2012; Yu et al., 2012), *Zymomonas mobiles* (Hayashi et al., 2012; Letti et al., 2012) and even *Escherichia coli* (Zhou et al., 2008; Manow et al., 2012) have been employed for ethanol production. As an alternative, photosynthetic cyanobacteria have been recently engineered by various synthetic biology tools into an “autotrophic microbial cell factory” to produce biofuels and fine chemicals directly from CO_2_ using solar energy (Angermayr et al., 2009; Ducat et al., 2011; Ruffing, 2011; Machado and Atsumi, 2012; Oliver and Atsumi, 2014), which provides a complementary approach to the above heterotrophic microorganisms. In a recent study, by systematic evaluation and selection of alcohol dehydrogenase (*adh*) genes from different cyanobacterial sources and optimization of culturing conditions, Gao et al. (2012) engineered a synthetic *Synechocystis* strain able to produce ethanol levels of 212 mg/L per day and 5.50 g/L in 26 days (Gao et al., 2012). Although significant progress has been made, current ethanol productivity in cyanobacterial systems is still largely lagging behind the yeast systems (Antoni et al., 2007). Recent studies have suggested that one of the crucial factors responsible for the low ethanol productivity of cyanobacterium cells could be their very low tolerance toward ethanol (Dunlop, 2011; Jin et al., 2014). For example, in the model cyanobacterium *Synechocystis* sp. PCC 6803 (hereafter *Synechocystis*), the cell growth was arrested by up to 50%, and started to aggregate under 1.5% (*v/v*) ethanol (Qiao et al., 2012). In another study, Kamarainen et al. (2012) also evaluated tolerance of the cyanobacterial hosts to various biofuels, and found that the *Synechocystis* growth was less than 50% of the control in BG11 medium supplemented with 2.0 g/L ethanol (Kamarainen et al., 2012). The studies suggested that it is necessary to adopt engineering strategies to ethanol tolerance in cyanobacterial systems.

Many organic solvents are toxic to microorganisms. The toxicity of organic solvents depends on its log *Pow* (the partition coefficient between n-octanol and water) and final concentration of accumulation in the cell which contribute to increase permeability of the membrane. Readily water-miscible organic solvents such as ethanol, toxicity correlates directly with hydrophobicity (Ingram, 1981). In addition, ethanol has been shown to affect the proton motive force (Cartwright et al., 1987), and to increase leakage of metabolites from cells.

A membrane transport protein (or simply transporter) is a membrane protein involved in the movement of ions, small molecules, or macromolecules, such as another protein, across a biological membrane. Protein transporters for small molecules have been suggested as an important mechanism against ethanol toxicity (Ding et al., 2009; Stanley et al., 2010; Foo et al., 2014). For example, studies showed that the FPS1 gene, encoding the plasma membrane aquaglyceroporin that can facilitate transmembrane transport of small-uncharged molecules like polyols and urea, was up-regulated in response to ethanol exposure in yeast (Aguilera et al., 2006; Teixeira et al., 2009). Similarly, a recent functional genomics study with *E. coli* under exogenous *n*-butanol stress also found that transporters were among the most regulated functional components (Rutherford et al., 2010). Another recent study, involving heterologous expressing of 43 transporters in *E. coli*, showed that selected transporter could improve cell survival under stress of biofuels such as geraniol and limonene; however, none of the transporters improved tolerance for *n*-butanol and isopentanol (Dunlop et al., 2011). Nevertheless, these early studies suggested that utilization of transporters could be one of the approaches for engineering tolerance and biofuels production strains (Ding et al., 2009; Stanley et al., 2010; Dunlop et al., 2011).

Transporters have been reported for roles against many types of environmental stresses in cyanobacteria, such as arsenate resistance in *Anabaena variabilis* (Thiel, 1988), Cu^2+^ resistance in *Nostoc calcicola* (Verma and Singh, 1991), salinity stress in *Synechococcus* sp. PCC 7942 and *Synechocystis* sp. PCC 6803 (Nomura et al., 1995; Mikkat et al., 1996), acid stress in *Synechocystis* sp. PCC 6803 (Tahara et al., 2012), and heavy metals in filamentous *Oscillatoria brevis* (Tong et al., 2002). Among all transporters in *Synechocystis*, ATP binding cassette (ABC) transporters residing in the inner membrane that are involved in the transport of a wide variety of substrates at the expense of ATP hydrolysis are the most common and well-characterized type (Davidson et al., 2008). For example, the *slr1295*, *slr0513*, *slr0327*, and *sll1878* genes encoding polypeptides of an ABC-type ferric iron transporter that played a major role in iron acquisition have been identified (Katoh et al., 2001). However, so far no transporter involved in ethanol resistance has ever been reported in any cyanobacterial species.

In this study, to discover transporters related to ethanol tolerance, we constructed a mutant library of transporter genes in *Synechocystis*, and then screened them for tolerance changes under ethanol stress. The efforts led to the discovery of the *slr0982* gene that may be involved in tolerance against ethanol stress in *Synechocystis*. In addition, to further explore the ethanol tolerance mechanism mediated by transporter Slr0982, we applied a metabolomics approach to comparatively analyze the differential cellular responses between the *Synechocystis* wild type strain and the Δ*slr0982* mutant (Wang et al., 2013). The study provides important findings for better understanding of the tolerance mechanism to ethanol stress in *Synechocystis*.

## Materials and methods

### Bacterial growth conditions and ethanol treatment

*Synechocystis* sp. PCC 6803 and the knockout mutants constructed in this study were grown in BG11 medium (pH 7.5) under a light intensity of approximately 50 μmol photons m^−2^ s^−1^ in an illuminating incubator of 130 rpm at 30°C (HNY-211B Illuminating Shaker, Honour, China) (Qiao et al., 2012). Cell density was measured on a UV-1750 spectrophotometer (Shimadzu, Japan) at OD_730_ or on an ELx808 Absorbance Microplate Reader (BioTek, Winooski, VT, USA) at OD_630_. For growth and ethanol treatment, 40 μL fresh cells at OD_630_ of 0.2 collected by centrifugation and were then inoculated into 200 μL of BG11 liquid medium in 96-well cultivation plates. Ethanol at a final concentration was added at the beginning of cultivation. The 96-well cultivation plates were fixed in the shaker and measured directly on ELx808 Absorbance Microplate Reader at OD_630_ every 12 h. To ensure accuracy of finding, the mutants with differential growth patterns under ethanol stress were then confirmed by growth in 250-mL flasks, in which 10 mL fresh cells collected by centrifugation and were inoculated into 50 mL of BG11 liquid medium in a 250 mL flask. Culture samples (1 mL) were taken and measured at both OD_730_ and OD_630_ every 12 h. Growth experiments were repeated at least three times to confirm the growth patterns.

### Construction and analysis of knockout mutants

A fusion PCR based method was employed for the construction of gene knockout fragments (Wang et al., 2002). Briefly, for the gene target selected, three sets of primers were designed to amplify a linear DNA fragment containing the chloramphenicol resistance cassette (amplified from a plasmid pACYC184) with two flanking arms of DNA upstream and downstream of the target gene. The PCR strategy of mutant construction was shown in Supplementary Figure S1. The linear fused PCR amplicon was used directly for transformation into *Synechocystis* by natural transformation. The chloramphenicol-resistant transformants were obtained and passed several times on fresh BG11 plates supplemented with 10 μg/mL chloramphenicol to achieve complete chromosome segregation. The successful knockout mutants were confirmed by colony PCR and sequencing analysis. PCR primers for mutant construction and validation were listed in Supplementary Table S1. Comparative growth analysis of the wild-type *Synechocystis* and the mutants were performed in 250 mL flasks each with 50 mL BG11 medium of normal or ethanol.

### Flow cytometric analysis

To reveal cell morphology differences, flow cytometric analysis was performed on a Calibur fluorescence-activated cell sorting (FACS) cytometer (Becton Dickinson) with the following settings: forward scatter (FCS), E00 log; side scatter, 400 V. Control and ethanol-treated cells were harvested at 24, 48, and 72 h, respectively, washed twice with phosphate buffer (pH 7.2) (Sigma-Aldrich), and then resuspended in the same phosphate buffer to a final OD_580_ of 0.3 (approximately 1.5 × 10^7^ cells mL^−1^). A total of 5 × 10^4^ cells were used for each analysis according to the method by Marbouty et al. (2009). Data analysis was conducted using the CellQuest software, version 3.1 (Becton Dickinson).

### Complementation of *slr0982* in the Δ*slr0982* mutant

Gene expressing vector pXT37b was kindly provided by Dr. Xuefeng Lu of Qingdao Institute of Bioenergy and Bioprocess Technology of Chinese Academy of Sciences (Tan et al., 2011). The promoter of plastocyanin (P_petE_) was first replaced by that of phycocyanin beta chain (P_cpcB_) in this study. The ORF of *slr0982* was subcloned into *Nde*I/*Xho*I site of the modified pXT37b, resulting in pXT-*slr0982*. The primers sequences for *slr0982* cloning were 5′-CGCCATATGATGTCTGATACAGTCATTCGAGTGG-3′ and 5′-CCGCTCGAGTCATGCAATTTTCTCCACATTCCAG-3′. The pXT-*slr0982* was introduced back into the Δ*slr0982* mutant by natural transformation. The *slr0982*-complementation strain was named *Δslr0982/*pXT-*slr0982*. To achieve complete chromosome segregation, *Δslr0982/*pXT-*slr0982* was passed several times on fresh BG11 plates supplemented with 10 μg/mL spectinomycin. Homologous integration of the expressing cassette and complete segregation were confirmed by PCR analysis.

### LC-MS based metabolomics analysis

*(i) Sample quenching, extraction, and preparation*: All chemicals used for LC-MS metabolomic analyses were obtained from Sigma-Aldrich (Taufkirchen, Germany). Cells were collected by centrifugation at 8000 × *g* for 8 min at room temperature (Eppendorf 5430R, Hamburg, Germany). The cell samples were quenched and extracted rapidly with 900 μL of 80:20 methanol/H_2_O (−80°C) and then frozen in liquid nitrogen. The samples were then frozen-thawed three times to release metabolites from the cells. The supernatant was collected after centrifugation at 15,000 × *g* for 5 min at −4°C and then stored at −80°C. The remaining cell pellets were re-suspended in 500 μL of 80:20 methanol/H_2_O (−80°C) and the above extraction process was repeated. The supernatant from the second extraction was pooled with that from the first extraction and stored at −80°C until LC-MS analysis (Bennette et al., 2011); (*ii*) *LC-MS analysis*: The chromatographic separation was achieved with a SYnergi Hydro-RP (C18) 150 mm × 2.0 mm I.D., 4 μm 80 Å particles column (Phenomenex, Torrance, CA, USA) at 40°C. Mobile phase A (MPA) is an aqueous 10 mM tributylamine solution with pH 4.95 adjusted with acetic acid and Mobile phase B (MPB) is 100% methanol of HPLC grade (Darmstadt, Germany). The optimized gradient profile was determined as follows: 0 min (0% B), 8 min (35% B), 18 min (35% B), 24 min (90% B), 28 min (90% B), 30 min (50% B), 31 min (0% B). A 14-min post-time equilibration was employed, bringing total run-time to 45 min. Flow rate was set as a constant 0.2 mL/min (Park et al., 2011). LC-MS analysis was conducted on an Agilent 1260 series binary HPLC system (Agilent Technologies, Waldbronn, Germany) coupled to an Agilent 6410 triple quadrupole mass analyser equipped with an electrospray ionization (ESI) source. Injected sample volume for all cases was 10 μL; capillary voltage was 4000 V; and nebulizer gas flow rate and pressure were 10 L/min and 50 psi, respectively. Nitrogen nebulizer gas temperature was 300°C. The MS was operated in negative mode for multiple reaction monitoring (MRM) development, method optimization, and sample analysis. Data were acquired using Agilent Mass Hunter workstation LC/QQQ acquisition software (version B.04.01) and chromatographic peaks were subsequently integrated *via* Agilent Qualitative Analysis software (version B.04.00); (*iii*) *Targeted metabolite analysis*: a total of 24 metabolites were selected for LC-MS based targeted metabolite analysis in this study as described previously (Su et al., 2014). The standard compounds for these 24 metabolites were purchased from Sigma, and their MS and MS/MS experimental parameters were optimized with the mix standard solution. All metabolomics profile data was first normalized by the internal control and the cell numbers of the samples, standardized by average, log2 transformed and then subjected to Principal Component Analysis (PCA) using software SIMCA-P 11.5 (Laiakis et al., 2010). Heatmap were created using MultiExperiment Viewer software available publically at http://www.tm4.org/.

### GC-MS based metabolomics analysis

All chemicals used for metabolome isolation and GC/MS analyses were obtained from Sigma-Aldrich (Taufkirchen, Germany). For metabolomic analysis, cells of the wild type and the Δ*slr0982* mutant were collected from control and ethanol-stressed cultures at 24, 48, and 72 h, respectively. For each sample, cells equivalent to ~10^8^ cells, were collected by centrifugation at 8000 × *g* for 10 min at 4°C (Eppendorf, Hamburg, Germany). The cell pellets were immediately frozen in liquid nitrogen and then stored at −80°C before use. The metabolomic analysis protocol included: (*i*) *Metabolome extraction*: cells were re-suspended in 1.0 mL cold 10:3:1 (*v/v/v*) methanol: chloroform: H_2_O solution (MCW), and frozen in liquid nitrogen and thawed for five times. Supernatants were collected by centrifugation at 14000 × *g* for 3 min at 4°C. To normalize variation across samples, an internal standard (IS) solution (100 μg/mL U-13C-sorbitol, 10 μL) was added to 100 μL supernatant in a 1.5-mL microtube before it was dried by vacuum centrifugation for 2–3 h (4°C). (*ii*) *Sample derivatization*: derivatization was conducted according to the two-stage technique described by Roessner et al. (2001). The samples were dissolved in 10 μL methoxyamine hydrochloride (40 mg/mL in pyridine) and shaken at 30°C for 90 min, then were added with 90 μL N-methyl-N-(trimethylsilyl) trifluoroacetamide (MSTFA) and incubated at 37°C for 30 min to trimethylsilylate the polar functional groups. The derivate samples were collected by centrifugation at 14000 × *g* for 3 min before GC/MS analysis. (*iii) GC-MS analysis*: analysis was performed on a Agilent 7890A Gas Chromatograph (GC) coupled to a Agilent MSD 5975 system (Agilent Technologies, Inc., Santa Clara, CA, USA) equipped with a HP-5MS capillary column (30 m × 250 mm id). 2 μL derivatized sample was injected in splitless mode at 230°C injector temperature. The GC was operated at constant flow of 1.0 mL/min helium. The temperature program started isocratic at 45°C for 2 min, followed by temperature ramping of 5°C/min to a final temperature of 280°C, and then held constant for additional 2 min. The range of mass scan was m/z 38–650. *iv*) *Data processing and statistical analysis*: The mass fragmentation spectrum was analyzed using the Automated Mass Spectral Deconvolution and Identification System (AMDIS) (Stein, 1999) to identify the compounds by matching the data with Fiehn Library (Fiehn, 2002) and the mass spectral library of the National Institute of Standards and Technology (NIST). Peak areas of all identified metabolites were normalized against the internal standard and the acquired relative abundances for each identified metabolite were used for future data analysis. All metabolomics profile data was first normalized by the internal control and the cell numbers of the samples, log2 transformed, and then subjected to PCA using software SIMCA-P 11.5 (Laiakis et al., 2010).

### Quantitative real-time RT-PCR analysis

The identical cultures used for metabolomics analysis were also used for RNA isolation and quantitative real-time RT-PCR (RT-qPCR) analysis of gene expression. RT-qPCR analysis was performed as described previously (Kloft et al., 2005; Wang et al., 2012). Quantification of gene expression was determined according to standard process of RT-qPCR that used serial dilutions of known concentration of chromosome DNA as template to make a standard curve. The 16s rRNA was used as an internal control. Three technical replicates were performed for each gene. Data analysis was carried out using the StepOnePlus analytical software (Applied Biosystems, Foster City, CA). Briefly, the amount of relative gene transcript was normalized by that of 16s rRNA in each sample (mutant or wild type), and the data presented were ratios of the amount of normalized transcript in the treatment between the mutant and the wild type. The gene ID and their related primer sequences used for real-time RT-qPCR analysis were also listed in Supplementary Table S1.

### WGCNA correlation network construction

Correlation network was created from the metabolomic data set, first by calculating weighted *Pearson* correlation matrices corresponding to metabolite abundance, and then by following the standard procedure of WGCNA to create the networks (Zhang and Horvath, 2005; Langfelder and Horvath, 2008; Wang et al., 2013). Briefly, weighted correlation matrices were transformed into matrices of connection strengths using a power function. These connection strengths were then used to calculate topological overlap (TO), a robust and biologically meaningful measurement that encapsulates the similarity of two metabolites' correlation relationships with all other metabolites in the network. Hierarchical clustering based on TO was used to group metabolites with highly similar correlation relationships into modules. Metabolite dendrograms were obtained by average linkage hierarchical clustering, while the color row underneath the dendgram showed the module assignment determined by the Dynamic Tree Cut of WGCNA. The network for each module was generated with the minimum spanning tree with dissimilarity matrix from WGCNA. The modules with correlation *r* > 0.5 or *r* < −0.5, and *p*-value less than 0.05 were extracted for further investigation. Hub metabolites were screened by high connectivity with other metabolites (≥5) in the modules strongly associated with genotypes based on correlation coefficient *r* > 0.5 or *r* < −0.5.

### Pathway enrichment analysis

Metabolic pathway enrichment analysis of the responsive metabolites was conducted using the information from the KEGG (Kyoto Encyclopedia of Genes and Genomes) database according to the following formula (Su et al., 2014):

$$P=1-\sum_{i\;=\;0}^{m\;-\;1}\frac{\left(\begin{array}{c} M\\ i \end{array}\right)\left(\begin{array}{c} N-M\\ n-i \end{array}\right)}{\left(\begin{array}{c} N\\ n \end{array}\right)}$$

*N* is the number of all metabolites within all KEGG pathways, *M* is the number of metabolites within a given KEGG pathway, *n* is the number of the responsive metabolites within all KEGG pathways, *m* is the number of the responsive metabolites within a given KEGG pathway. A *p*-value less than 0.05 was used as a cutoff for enriched KEGG pathways.

## Results and discussion

### Screening for ethanol sensitivity in transporter mutant library

**Figure 1:**
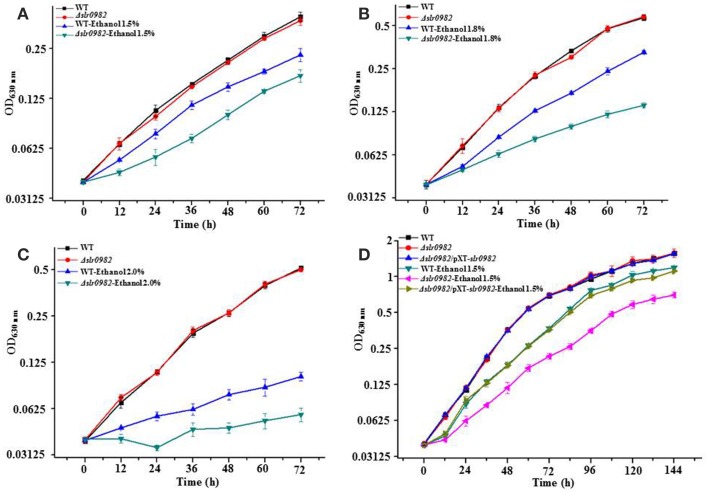
**Growth time curves of the wild type, the Δ*slr0982* mutant and *Δslr0982/*pXT-*slr0982* in BG11 media with or without ethanol. (A)** and **(D)**, 1.5% (*v/v*); **(B)** 1.8% (*v/v*); **(C)** 2.0% (*v/v*).

A survey showed that at least 387 putative transporter-encoding genes were present in the *Synechocystis* genome (Kaneko et al., 1996). In this study, to uncover transporter genes involved in ethanol tolerance in *Synechocystis*, we excluded all transporter genes with well-characterized functions, such as bicarbonate or phosphate transporting. To this end, a total of 58 knockout mutants of transporter genes were constructed and the gene deletion was confirmed by PCR (Supplementary Table S1). To achieve complete segregation, positive colonies were passaged through several cultivations on solid BG11 plates supplied with gradually increased chloramphenicol, and the full integration into all chromosomes was also confirmed by PCR (Supplementary Figures S1A–M). After 12 passages under selective pressure, full segregation was achieved for 54 transporter genes except for the *Δslr0949*, *Δslr1224*, *Δslr1248*, and *Δsll1453* genes, suggesting that these transporter genes may carry essential roles for the cellular growth under the testing conditions. The mutants, in parallel with the wild-type *Synechocystis*, were then monitored for growth under various ethanol concentrations (i.e., 1.5, 1.8, and 2.0%, *v/v*) in 96-well cultivation plates. To ensure accuracy of the growth measurement, differential growth patterns under ethanol stress were also confirmed by growth in 250-mL flasks. The screening allowed the identification of Δ*slr0982* mutant, which was more sensitive to ethanol stress than the wild type. No significant tolerance change was found for other mutants under the tested condition. The full integration of Δ*slr0982* was shown in Supplementary Figure S1. As shown in Figure 1, in the normal BG11 medium, the Δ*slr0982* mutant grew equally well as the wild-type control, suggesting the knockout of *slr0982* gene didn't affect cell growth under normal growth condition. However, in the BG11 medium supplemented with ethanol, although growth of both the wild-type strain and the Δ*slr0982* mutant was reduced, growth of the Δ*slr0982* mutant was reduced more significantly when compared with its growth in the normal BG11 medium (Figures 1A–C). In addition, the results showed that the ethanol sensitivity of the Δ*slr0982* mutant seemed concentration-dependent, as more growth reduction was observed in the medium supplemented with 1.8% than with 1.5% ethanol (Figures 1A,B). Taken together, the results showed that the Δ*slr0982* mutant was more sensitive to ethanol stress than the wild-type cells, and the *slr0982* gene may be involved in ethanol tolerance in *Synechocystis*.

To further confirm the involvement of gene *slr0982* in ethanol tolerance, we constructed a *slr0982*-complementation strain in the Δ*slr0982* mutant, and the effect of the complementation was confirmed by comparatively measuring growth time-curve of relative strains. The results showed that in the normal BG11 medium, three strains grew almost equally; while in the BG11 medium with ethanol, the growth of *Δslr0982/*pXT-*slr0982* was almost fully recovered to that of wild type, and significantly better than the Δ*slr0982* mutant under the same ethanol stress, demonstrating that *slr0982* was involved in ethanol tolerance in *Synechocystis* (Figure 1).

The *slr0982* gene, also designated as *rfbB* gene, was annotated as O-antigen (OAg) transport gene in the Cyanobase genome database (Nakao et al., 2010). In a recent study, Fisher et al. (2013) studied the possible function of *slr0982* in *Synechocystis*, and the results showed no difference between OAg purified from the wild-type and the deletion mutant *slr0982*, although the bioinformatics inspection suggested the gene product *slr0982* appeared to function in OAg transport; furthermore, the results showed that exopolysaccharides (EPS) purified from the Δ*slr0982* mutant was altered in composition when compared to the wild type, suggesting that the *slr0982* gene may be involved in surface modification. In addition, analysis of the *slr0982* mutant showed that it was deficient in EPS export compared to wild-type, suggesting the Slr0982 transporter may be involved in exporting of EPS (Fisher et al., 2013). The results were consistent with early studies in yeast and *Z. mobilis* that the composition of cell membrane and cell wall can significantly influence ethanol tolerance (Hermans et al., 1991; Ding et al., 2009). Interestingly, *slr0982* seemed located within a putative operon, in which three hypothetical gene, *slr0978*, *slr0980*, and *slr0981* without any known function were located upstream, where several genes involved in polysaccharide metabolism, *slr0983* (glucose-1-phosphate cytidylyltransferase), *slr0984* (CDP-glucose 4,6-dehydratase), and *slr0985* (dTDP-4-dehydrorhamnose 3,5-epimerase) were located downstream of *slr0982*, respectively (Fisher et al., 2013). The results were also consistent with our previous RNA-seq transcriptomics analysis which showed that expression of *slr0983* was differentially regulated by ethanol exposure (Wang et al., 2012), further suggesting that the *slr0982* gene and the *slr0978*-*slr0985* gene cluster might be involved in ethanol tolerance.

### Analysing cell morphology of the wild-type and the Δ*slr0982* strain

**Figure 2:**
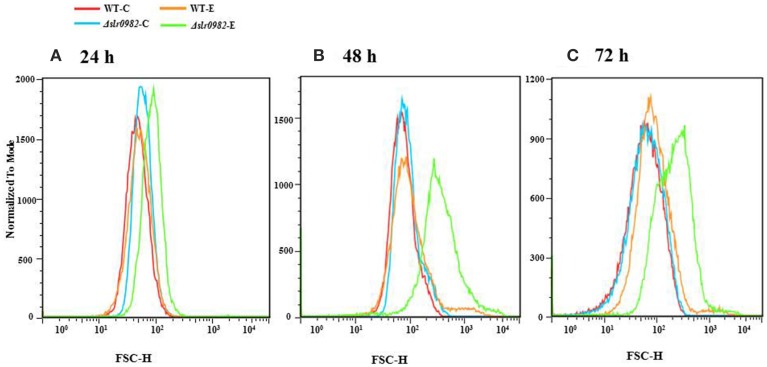
**Flow cytometric analysis of the wild type and the Δ*slr0982* mutant grown in the BG11 media with or without 1.5% (*v/v*) ethanol stress. (A)** 24 h; **(B)** 48 h; **(C)** 72 h. A total of 5 × 10^4^ cells were used for each analysis. Foward scatter (FSC) is related to cell surface area, which is directly related to cell size. And Y-axis was normalized pixel count. WT-C and WT-E: wild type grown with or without 1.5% (*v/v*) ethanol stress. Δ*slr0982*-C and Δ*slr0982*-E: the Δ*slr0982* mutant grown with or without 1.5% (*v/v*) ethanol stress.

Cell morphology of the wild-type *Synechocystis* and the Δ*slr0982* mutant under both control and ethanol-spiked conditions was also compared using microscopic inspection and flow cytometric analyses. Although no difference was observed with microscope, the flow cytometric analyses showed that the cell size of the Δ*slr0982* mutant was slightly increased under ethanol stress when compared with wild type. The size increase became more obvious after longer stress treatment at 48 and 72 h when compared with 24 h after ethanol stress (Figure 2). Many types of microbial cells are surrounded by thick exopolysaccharide chains, which are part of the network maintaining cell size, as it has been reported that altered EPS production occurred (i.e., less large EPS band while more small EPS band in the Δ*slr0982* mutant) (Fisher et al., 2013), so it is probably not surprising that the change of exopolysaccharides synthesis will affect cell size and cellular response to stress. For example, a glycogen excess *E. coli* mutant TR1-5 was found to exhibit effects on cell size and surface (adherence) properties (Romeo et al., 1993).

### Targeted LC-MS metabolomic analysis of the wild-type and the Δ*slr0982* strain

LC–MS based metabolomics analysis has been recently applied to studies of cyanobacterial metabolism (Bennette et al., 2011; Schwarz et al., 2013), due to its advantages toward chemically unstable metabolites, such as the redox active nucleotides (NADPH, NADH) and the hydrolytically unstable nucleotides (ATP, GTP, cAMP, PEP) that are crucial for all major metabolic pathways. Using an optimized protocol of sampling, chromatography and mass spectral detection (Wang et al., 2014b), the LC-MS metabolomic analysis allowed successful monitoring of the intracellular levels of 24 metabolites in *Synechocystis*, including intermediates in central carbon metabolism, cellular energy charge and redox poise (Bennette et al., 2011). We eventually established reproducible analyses for 24 key metabolites in the central metabolism in *Synechocystis*, including AcCOA, ADP, ADP-GCS, AKG AMP, ATP, COA, DHAP, FBP, F6P, FUM, GAP, G6P, GLU, NAD, NADH, NADP, NADPH, UDP-GCS, OXA, PEP, 3PG, R5P, and RiBP involved in the key metabolic pathways. Using them as references, a semi-quantitative characterization of all 24 metabolites from all *Synechocystis* cell samples is achieved. With the optimized LC-MS protocol, we collected the cells of the wild type and the Δ*slr0982* mutant grown in BG11 media (control) and ethanol 1.5% (*v/v*) at three time points (i.e., 24, 48, and 72 h). Each sample consisted of three biological replicates (Supplementary Table S2). To comparatively evaluate the effects of gene knockout on *Synechocystis* metabolism, we generated PCA score plots of metabolomic profiles of the wild-type strain and the Δ*slr0982* mutant through the growth time course under ethanol stress (Supplementary Figures S2A–C). Analysis of the score plots revealed obvious clustering patterns of three biological replicates for each sample, suggesting good analytical quality. In addition, the metabolomic profiles of the mutant can be visibly separated from those of the wild type at both control and ethanol stress conditions, suggesting good analytical resolution which can distinguish different metabolic status in the cells. Moreover, a slightly decreasing difference between the wild type and the Δ*slr0982* mutant under ethanol stress condition was observed, probably due to the fact that the cells started aging after a longer time of ethanol-stress treatment, so that metabolomic profiles of cells were more a reflection of cell aging rather than that of ethanol stress response.

**Figure 3:**
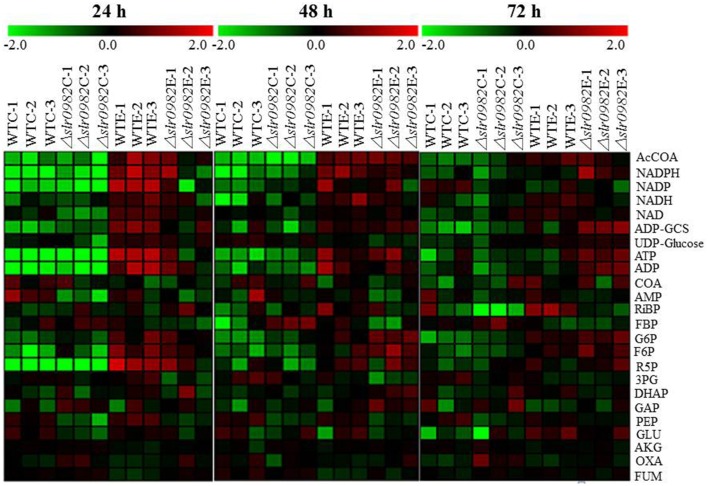
**Heat map analysis of LC-MS metabolomics**. The row displays metabolite and the column represents the samples. Metabolites significantly decreased were displayed in green, while metabolites significantly increased were displayed in red. The brightness of each color corresponded to the magnitude of the difference when compared with average value. WT-C and WT-E: wild type grown with or without 1.5% (*v/v*) ethanol stress. Δ*slr0982*-C and Δ*slr0982*-E: the Δ*slr0982* mutant grown with or without 1.5% (*v/v*) ethanol stress.

Heat maps were created for all 36 LC-MS metabolomic profiles from three time points (i.e., 24, 48, 72 h) (Figure 3). In the analysis, the ratio of a given metabolites was calculated between the concentration of the metabolite under a given condition and the average concentration of the metabolite in all samples at each time point. At 24 h, under control condition without ethanol stress, very similar metabolite abundance patterns were found for almost all metabolites between the wild type and the Δ*slr0982* mutant, consistent with their similar growth pattern in the normal BG11 media. However, when the wild type grown under control and ethanol stress was compared, the results showed that a majority of metabolites were found up-regulated under the ethanol stress condition, more significantly for AcCOA, NADPH, NADP, NADH, NAD, ADP-GCS, ATP, ADP, G6P, F6P, and R5P, which were key co-enzyme or bioenergetics molecules participating in many important metabolic pathways, such as glycolysis, pentose phosphate pathway and TCA cycle, and well-known to have crucial roles in stress response. As an example, it has been reported that NADPH as an important coenzyme participates in ethanol tolerance in yeast, where overexpression of NADPH-dependent alcohol dehydrogenase (ADH6) significantly increased the 5-hydroxymethylfurfural tolerance (Petersson et al., 2006). Glucose-6-phosphate dehydrogenase that catalyzes the conversion from G6P and NADP to 6-phospho-*D*-glucono-1,5-lactone and NADPH was responsive to radiation stress in *Synechococcus lividus* (Conter et al., 1987). Ribose-5-phosphate isomerase that catalyzes the conversion between ribose-5-phosphate (R5P) and ribulose-5-phosphate (Ru5P) was found differentially regulated under oxidative stress conditions in photosynthetic green alga *Chlamydomonas reinhardtii* (Zaffagnini et al., 2012). Sucrose-phosphate synthase (SpsA, Sll0045) that uses F6P as substrate to form sucrose 6-phosphate is a key enzyme to synthesize one major compatible solute, sucrose, against salt stress in *Synechocystis* (Curatti et al., 1998; Desplats et al., 2005; Klahn and Hagemann, 2011).

At 48 and 72 h, under control condition without ethanol stress, differences in terms of metabolite abundance patterns were observed between the wild type and the Δ*slr0982* mutant, suggesting that after a long time of ethanol stress treatment, cellular metabolism started to diverge even though their growth patterns were similar. In addition, such a difference seemed more obvious at 72 h than 48 h (Figure 3). In the wild type between control and ethanol stress conditions, similar patterns of increased abundances of key molecules were found in the cells under ethanol stress condition, although the increases seemed less significantly, especially at 72 h, which was probably due to the accelerated cell aging and death after longer time of ethanol stress. However, in the Δ*slr0982* mutant cells at 48 and 72 h, the abundances of AcCOA, ADP-GCS, ATP, ADP, G6P, F6P, and R5P were enhanced when compared with 24 h (Figure 3). Taken together, the LC-MS targeted metabolomic analysis suggested that the *slr0982* deletion could affect or delay the up-regulation of several key metabolites responsive to ethanol stress, which may eventually contribute to the tolerance loss in the Δ*slr0982* mutant (Figure 1).

To validate results from LC–MS analysis, 6 genes were selected for quantitative RT-PCR analysis; they are glucose-6-phosphate isomerase (*slr1349*), 6-phosphofructokinase (*sll0745*), phosphoribulokinase (*sll1525*), phosphopyruvate hydratase (*slr0752*), citrate synthase (*sll0401*) and glutamate dehydrogenase (*slr0710*). The genes were selected because their enzymatic substrates or products were differentially regulated between the *slr0982* mutant and the control under ethanol stress condition, as revealed by the metabolomic analysis (Figure 3). The gene ID and their related primer sequences used for real-time RT-qPCR analysis were listed in Supplementary Table S1. Comparative RT-qPCR analysis showed these genes were down-regulated 1.5–2.6 folds under ethanol stress in the mutant when compared with the wild type (Table 1). In general, good agreement between the gene expression analysis and the LC–MS metabolomic analysis was observed: down-regulation of *slr1349* and *sll0745* genes was consistent with the decreased abundance of FBP, while down-regulation of *sll1525*, *slr0752*, and *slr0710* was consistent with the decreased abundances of RiBP, PEP and Glu, respectively.

**Table 1:** **RT-qPCR analysis of selected genes**.

**Gene ID**	**Gene description**	**Comparision**	**RT-qPCR ratio**
*slr1349*	glucose-6-phosphate isomerase	Δ*slr0982*-ethanol vs. WT-ethanol	−1.771 ± 0.052
*sll0745*	6-phosphofructokinase	Δ*slr0982*-ethanol vs. WT-ethanol	−2.168 ± 0.229
*sll1525*	Phosphoribulokinase	Δ*slr0982*-ethanol vs. WT-ethanol	−1.452 ± 0.072
*slr0752*	phosphopyruvate hydratase	Δ*slr0982*-ethanol vs. WT-ethanol	−2.613 ± 0.282
*sll0401*	citrate synthase	Δ*slr0982*-ethanol vs. WT-ethanol	−2.090 ± 0.297
*slr0710*	glutamate dehydrogenase	Δ*slr0982*-ethanol vs. WT-ethanol	−1.773 ± 0.323

### Untargeted GC-MS metabolomic analysis of the wild-type and the Δ*slr0982* strain

**Figure 4:**
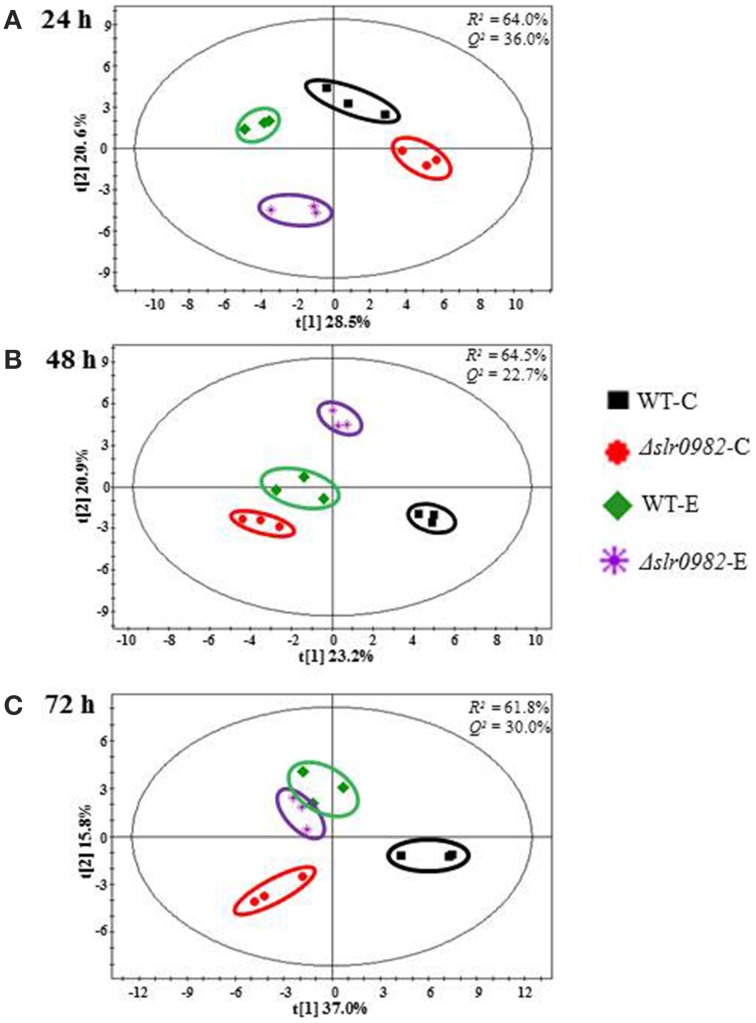
**PCA plot analysis of GC-MS metabolomic profiles. (A)** 24 h; **(B)** 48 h; **(C)** 72 h. Each dot represents one biological sample, while the dots of the same color are biological replicates. Four groups of samples, the wild type and the Δ*slr0982* mutant grown in the BG11 media with or without 1.5% (*v*/*v*) ethanol stress are indicated by different colors, and each group is indicated by a circle. WT-C and WT-E: wild type grown with or without 1.5% (*v/v*) ethanol stress. Δ*slr0982*-C and Δ*slr0982*-E: the Δ*slr0982* mutant grown with or without 1.5% (*v/v*) ethanol stress.

Following the optimized extraction protocol established previously (Krall et al., 2009), the cells of the wild type control and the Δ*slr0982* mutant grown in BG11 media with and without 1.5% (*v/v*) ethanol stress were collected at three time points (i.e., 24, 48, and 72 h) and the further MS analysis allowed the chemical classification of a total 48 metabolites from *Synechocystis*, including various amino acids, sugars, and organic acids detected in almost all replicate samples (Supplementary Table S3). Score plots of PCA were generated to compare the mutant and the wild type in different conditions at three time points (i.e., 24, 48, and 72 h). The time-series metabolomic data showed that: (*i*) clustering of the biological replicates for each sample was clearly observed, suggesting overall good quality of the GC-MS analysis; (*ii*) interestingly, although no difference was observed for the wild type and the Δ*slr0982* mutant in terms of cell growth in the control media, clustering analysis based on their intracellular metabolites showed that they were clearly separated even at 24 h (Figure 4), indicating that the metabolic difference between the wild type and the Δ*slr0982* mutant, consistent with the above results from the LC-MS metabolomics (Figure 3); (*iii*) through the time course from 24 to 72 h under ethanol stress condition, metabolomic profiles of the Δ*slr0982* mutant were gradually moving more close to those of the wild type, probably due to the increasing cell aging and death after long ethanol stress (Figure 4).

### WGCNA analysis of metabolomic profiles associated with the ethanol tolerance

To identify metabolic modules and hub metabolites related to ethanol tolerance in the Δ*slr0982* mutant, a correlation-based WGCNA analysis was applied to analysis to the GC-MS metabolomic datasets (Zhang and Horvath, 2005; Langfelder and Horvath, 2008). The network analysis was not applied to LC-MS metabolomic data due to their relatively small data size. In this study, we constructed unsigned networks using a GC-MS metabolomic data set consisted of 48 metabolites, and then localized the correlated metabolites into various metabolic modules. In addition, the association of each distinguished metabolic module with mutant or ethanol stress treatment was determined. Setting a minimal number of metabolites in any module greater than 3, our WGCNA analysis showed that 6, 8, and 7 distinct metabolic modules can be detected within the metabolic networks at 24, 48, and 72 h, respectively (Supplementary Figure S3). Using a cutoff of correlation coefficient (*r* value) greater than 0.5 or less than −0.5, and their statistical confidence (*p*-values) less than 0.05, the analysis showed that a total of 4, 4, and 2 distinguished metabolic modules were highly associated with mutant or ethanol stress condition at 24, 48, and 72 h, respectively (Supplementary Figures S4A–C). At each time point, six modules (i.e., M1, M2, M3, M6, M8, and M9) were associated only with ethanol stress, three modules (i.e., M4, M5, and M7) were only associated with the knockout of the *slr0982* gene, and M10 module was associated with both the ethanol stress and the knockout of the *slr0982* gene, respectively. Metabolites included in each of the highly associated modules were presented in Table 2.

**Table 2:** Metabolites included in each of the highly associated modules^*^.

**Modules**	**Conditions**	**Time (h)**	**Association (*r*)**	***P***	**Metabolites**
M1	Ethanol	24	0.88	2.E-04	*L*-threonin, adenosine, capric acid, malonic acid, 2-amino-1-phenylethanol, 2-hydroxybutyric acid
M2	Ethanol	24	0.85	4.E-04	urea, 5-hydroxy-*L*-tryptophan, gluconic acid lactone
M3	Ethanol	24	0.69	1.E-02	*D*-allose, *DL*-isoleucine, oleic acid, heptadecanoic acid, linoleic acid, lauric acid, myristic acid, palmitic acid, stearic acid, 2-hydroxypyridine, palmitoleic acid
M4	*slr0982*	24	−0.74	6.E-03	*D*-(+)-galactose, *DL*-3,4-dihydroxyphenyl-glycol, succinic acid, *D*-glucose-6-phosphate, *L*-serine, *L*-norleucine, 3-hydroxypyridine, benzene-1,2,4-triol, phytol, *L*-(+)-lactic acid, spermidine, benzoic acid, porphine, sucrose, glycerol-1-phosphate, *L*-pyroglutamic acid, *D*-(+)-trehalose, *L*-glutamic acid
M5	*slr0982*	48	−0.88	2.E-04	malonic acid, *L*-threonine, palmitoleic acid
M6	Ethanol	48	0.65	2.E-02	urea, 2-hydroxypyridine, phytol, capric acid, succinic acid, caprylic acid, 3-hydroxypyridine, myristic acid, glycine, stearic acid
M7	*slr0982*	48	0.7	1.E-02	glycerol-1-phosphate, *D*-(+)-trehalose, 2-hydroxybutyric acid, *L*-norleucine, 2-amino-1-phenylethanol, palmitic acid, phosphoric acid
M8	Ethanol	48	−0.73	7.E-03	benzene-1,2,4-triol, *D*-allose, glycerol
M9	Ethanol	72	−0.98	9.E-09	malonic acid, glycolic acid, porphine
M10	*slr0982*	72	−0.67	2.E-02	sucrose, *D*-allose, benzene-1,2,4-triol, benzoic acid
	Ethanol	72	−0.66	2.E-02	

* *GC-MS metabolomic dataset was used for this analysis. The association of each distinguished metabolic module with mutant or ethanol stress treatment was determined*.

Using a criterion that at least two of its member metabolites identified from any given pathway, a pathway enrichment analysis was conducted and the results showed eight pathways were enriched in various distinct modules (Table 3). Among them, the results showed that “Tryptophan metabolism” (Map00380) was enriched with statistical significance *p*-values less than 0.05 in ethanol-associated module M2. Enrichment of “Tryptophan metabolism” in ethanol-associated modules was consistent with early studies in *E. coli* and *Saccharomyces cerevisiae*, in which tryptophan biosynthetic pathway was previously found up-regulated in the ethanol-tolerant *E. coli* strains by a microarray analysis, and supplementation of tryptophan to the culture medium increased the specific growth rate of *E. coli* under ethanol stress (Horinouchi et al., 2010), and overexpressing tryptophan biosynthesis genes in yeast resulted in a stress tolerance to 5% ethanol (Hirasawa et al., 2007). In addition, “Biosynthesis of unsaturated fatty acids” (Map01040) was enriched in ethanol-associated module M3, and “Fatty acid biosynthesis” (Map00061) was enriched in both ethanol-associated module M3 and M6, consistent with the well-defined roles of fatty acid, especially unsaturated fatty acids in membrane modification against ethanol stress in various microbes (Heipieper and De Bont, 1994; Chiang et al., 2008). One well-described change is the shift from *cis* to *trans* unsaturated fatty acids to decrease membrane fluidity, resulting in a corresponding increase in solvent tolerance (Dunlop, 2011). In a previous study with cyanobacteria, the acyl-lipid desaturase (*desA*) gene from *Synechocystis* was expressed in prokaryotic *E. coli* and eukaryotic *Solanum tuberosum* cells, which led to an enhanced cold tolerance due to increased unsaturated fatty acid concentration in their lipids (Amiri et al., 2010). Our previous RNA-seq transcriptomics analysis also showed that *slr1350* encoding acyl-lipid desaturase was up-regulated by ethanol exposure in *Synechocystis* (Wang et al., 2012).

**Table 3:** Pathway enrichment analysis of metabolites associated with the ethanol stress condition^*^.

**Pathways**	**KEGG pathway ID**	**Modules associated**	***p*-values (over represented)**	**Number of associated metabolites in given KEGG pathway**	**Number of all metabolites in given KEGG pathway**	**Metabolites involved**
Tryptophan metabolism	Map00380	M2	0.0400	1	1	5-hydroxy-*L*-tryptophan
Biosynthesis of unsaturated fatty acids	Map01040	M3	0.0120	4	4	palmitic acid, stearic acid, oleic acid, linoleic acid
Fatty acid biosynthesis	Map00061	M3	0.0152	5	7	palmitic acid, stearic acid, oleic acid, myristic acid, lauric acid
Biosynthesis of secondary metabolites	Map01110	M4	0.0384	10	15	benzoic acid, succinic acid, *D*(+) trehalose, *L*-glutamic acid, *D*-glucose-6-phosphate, *L*-norleucine, *D* (+) galactose, *L*-serine, spermidine, sucrose
Fatty acid biosynthesis	Map00061	M6	0.0117	4	7	capric acid, caprylic acid, myristic acid, stearic acid
Chloroalkane and chloroalkene degradation	Map00625	M9	0.0428	1	1	glycolic acid
Benzoate degradation	Map00362	M10	0.0069	2	2	benzoic acid, benzene-1,2,4-triol
Aminobenzoate degradation	Map00627	M10	0.0069	2	2	benzoic acid, benzene-1,2,4-triol

* *GC-MS metabolomic dataset was used for this analysis*.

Enrichment analysis showed that “Biosynthesis of secondary metabolites” (Map01110), “Chloroalkane and chloroalkene degradation” (Map00625), “Benzoate degradation” (Map00362) and “Aminobenzoate degradation”(Map00627) were enriched with statistical significance *p*-values less than 0.05 in module M4, M9, M10, and M10, respectively (Table 2). The “Biosynthesis of secondary metabolites” pathway contains many metabolites known to be related to stress conditions, such as *D*(+) trehalose involved in salt stress in cyanobacteria (Hagemann, 2011), ethanol resistance in *E. coli* (Wang et al., 2013) and *S. cerevisiae* (Zheng et al., 2013; Wang et al., 2014a), and *L*-isoleucine and *L*-glutamic acid involved in ethanol tolerance in *E. coli* (Horinouchi et al., 2010; Wang et al., 2013). “Benzoate degradation” (Map00362) and “Aminobenzoate degradation” (Map00627) were enriched due to the same two metabolites, benzoic acid and benzene-1,2,4-triol. However, their roles in ethanol tolerance are still unclear.

**Figure 5:**
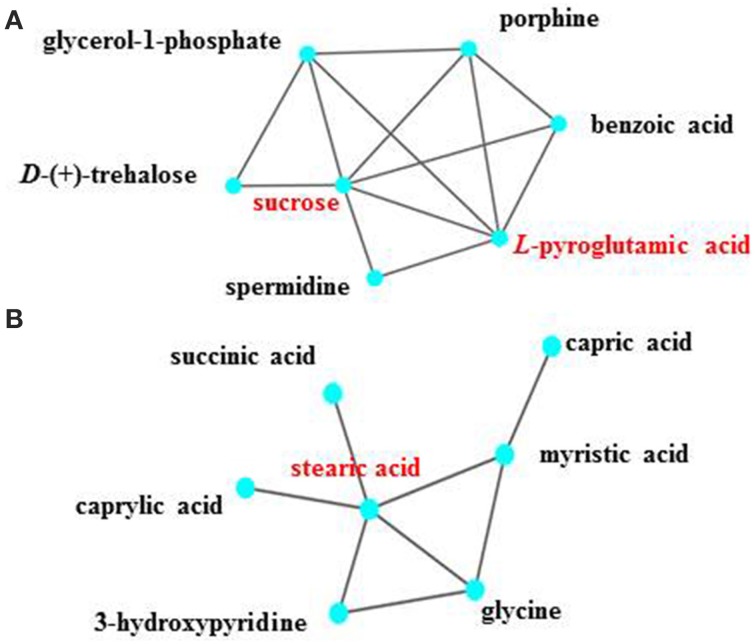
**The hub metabolites and their metabolic profiles as represented by the node and edge graph. (A)** Sucrose and *L*-pyroglutamic acid in the module M4 at 24 h; **(B)** Stearic acid in the module M6 at 48 h.

Within metabolic networks, hub metabolites that are involved in a high number of reactions are typically biologically important (Pfeiffer et al., 2005). Assuming a cutoff of connectivity greater than 5 in the networks as hub metabolites, a few hub metabolites were identified from the metabolic network constructed by the WGCNA, including sucrose and *L*-pyroglutamic acid in the module M4 at 24 h and stearic acid in the module M6 (Figure 5). While M6 was associated with only ethanol stress, the role of stearic acid along with several connected fatty acid may be associated with membrane modification against ethanol stress (Dunlop, 2011). M4 was associated with the *slr0982* knockout event (Table 2). As discussed above, sucrose was well studied as compatible solutes against salt stress in cyanobacteria (Hagemann, 2011). As osmoprotectants, pyroglutamic acid was found to accumulate in response to salt stress and function as an osmoprotectant along with sucrose, in the halotolerant methanotroph *Methylobacter alkaliphilum* (Trotsenko and Khmelenina, 2002). In addition, these two compouds were also found to be involved in ethanol tolerance in *Synechocystis* (Zhu et al., 2015).

## Conclusions

By constructing and screening a mutant library of 58 transporter-encoding genes, a gene encoding an ABC transporter, *slr0982*, was identified to be involved in ethanol tolerance in *Synechocystis*. As the first ethanol-tolerance related transporter found in cyanobacteria, the gene could be valuable as a target of ethanol tolerance engineering for high-efficiency production in cyanobacterial systems. Although no evidence showed that Slr0982 is directly involved in the export of ethanol, the analysis suggested that it may affect transporting of other molecules important to the function and maintance of cytoplasmic membrane or extracellular structure.

To further decipher the ethanol tolerance mechanism mediated by transporter Slr0982, comparative LC-MS and GC-MS based metabolomic analyses were applied to determine the variation of intracellular intermediates in the wild type and the Δ*slr0982* mutant grown in BG 11 media with or without ethanol stress. The PCA and WGCNA analyses were used for analyzing the metabolomic data set in order to identify specific metabolites responsive to ethanol stress between mutant and wild type. The results showed that metabolites such as AcCOA, ADP-GCS, ATP, ADP, G6P, F6P, and R5P, and metabolic modules such as “Tryptophan metabolism,” “Biosynthesis of unsaturated fatty acids” and “Biosynthesis of secondary metabolites” might be related to ethanol tolerance in cyanobacterium *Synechocystis*, and further analysis the mechanisms that these metabolites and modules may represent could be valuable for tolerance in *Synechocystis*. Taken together, this study provided new insights into ethanol tolerance in cyanobacterium *Synechocystis*.

## Author contributions

LC and WZ conceived of the study. YZ, LC, and WZ drafted the manuscript. YZ carried out the mutant construction, overexpression, phenotypic and RT-PCR analyses. GP carried out the metabolomic data analysis. XN and XZ carried out the LC-MS analysis. MS and XZ carried out the GC-MS analysis. All authors read and approved the final manuscript.

### Conflict of interest statement

The authors declare that the research was conducted in the absence of any commercial or financial relationships that could be construed as a potential conflict of interest.

## Supplementary material

Supplementary Figure S1 **The schematic PCR strategy and valiation of the mutants**. Agarose gel electrophoresis in A–M were the PCR validation data. The same row, the same group. F1, R3 and internal primers (Supplementary Table S1) were used for colony PCR validation. Lane M: 1 kb DNA markers. The other lanes: The chromosomal DNA of WT or mutant was used as PCR template.

Supplementary Figure S2 **PCA plot analysis of LC-MS metabolomic profiles. (A)** 24 h; **(B)** 48 h; **(C)** 72 h. Each dot represents one biological sample, while the dots of the same color are biological replicates. Four groups of samples, the wild type and the Δ*slr0982* mutant grown in the BG11 media with or without 1.5% (*v*/*v*) ethanol stress are indicated by different colors, and each group is indicated by a circle. WT-C and WT-E: wild type grown with or without 1.5% (*v/v*) ethanol stress. Δ*slr0982*-C and Δ*slr0982*-E: the Δ*slr0982* mutant grown with or without 1.5% (*v/v*) ethanol stress.

Supplementary Figure S3 **Module-trait relationships of GC-MS metabolic profiles of the *Synechocystis* during ethanol-treated. (A)** 24 h; **(B)** 48 h; **(C)** 72 h. Setting a minimal number of metabolites in any module greater than 3, WGCNA analysis showed that 6, 8, and 7 distinct metabolic modules with mutant or ethanol stress can be detected within the metabolic networks at 24, 48, and 72 h, respectively. Metabolites in the module significantly decreased were displayed in green, while metabolites in the module significantly increased were displayed in red.

Supplementary Figure S4 **Weighted correlation network analysis (WGCNA) of GC-MS metabolic profiles of the *Synechocystis* during ethanol-treated. (A)** 24 h; **(B)** 48 h; **(C)** 72 h. The distinct modules identified at each time point were indicted by the clustering patterns of the red color squares along the diagonal inside the plots. The modules highly associated with any given ethanol stress (*r* > 0.5 or *r* < −0.5 and *p* < 0.05) were indicated with their metabolites in corresponding colors with their modules, and the correlation coefficients and *p*-values were shown in Table 2.

## Abbreviations

AcCOA: Acetyl coenzyme A
ADP: Adenosine 5′-diphosphate
ADP-GCS: Adenosine-5′-diphosphoglucose
AKG: α-Ketoglutaric acid
AMP: Adenosine 5′-monophosphate
AMDIS: Automated Mass Spectral Deconvolution and Identification System
ATP: Adenosine 5′-triphosphate
COA: Coenzyme A hydrate
DHAP: Dihydroxyacetone phosphate
FBP: *D*-Fructose 1,6-bisphosphate
F6P: *D*-Fructose 6-phosphate
FUM: Sodium fumarate dibasic
GAP: *DL*-Glyceraldehyde 3-phosphate
GC-MS: Gas Chromatography-Mass Spectrometry
GLU: *L*-Glutamic acid
G6P: *D*-Glucose 6-phosphate
LC-MS: Liquid Chromatography–Mass Spectrometry
MSTFA: N-methyl-N-(trimethylsilyl) trifluoroacetamide
NAD: α-Nicotinamide adenine dinucleotide
NIST: National Institute of Standards and Technology
OXA: Oxaloacetic acid
PCA: Principal Component Analysis
PCR: Polymerase Chain Reaction
PEP: Phospho(enol)pyruvic acid
3PG: *D*-(-)-3-Phosphoglyceric acid
RiBP: *D*-Ribulose 1,5-bisphosphate
R5P: *D*-Ribose 5-phosphate
RT-qPCR: Real-time Quantitative Polymerase Chain Reaction
UDP-GCS: Uridine 5′-diphosphoglucose
WGCNA: Weighted Correlation Network Analysis.
