# Supplementary material for: Identification of a transporter Slr0982 involved in ethanol tolerance in cyanobacterium Synechocystis sp. PCC 6803
Source: Front Microbiol. 2015 May 18;6:487. doi: 10.3389/fmicb.2015.00487 (PMC4440267; doi:10.3389/fmicb.2015.00487)
Supplement: Supplementary Figure S1 — The schematic PCR strategy and valiation of the mutants. Agarose gel electrophoresis in A–M were the PCR validation data. The same row, the same group. F1, R3 and internal primers (Supplementary Table S1) were used for colony PCR validation. Lane M: 1 kb DNA markers. The other lanes: The chromosomal DNA of WT or mutant was used as PCR template. [file Presentation1.PPTX]

## Slide 1
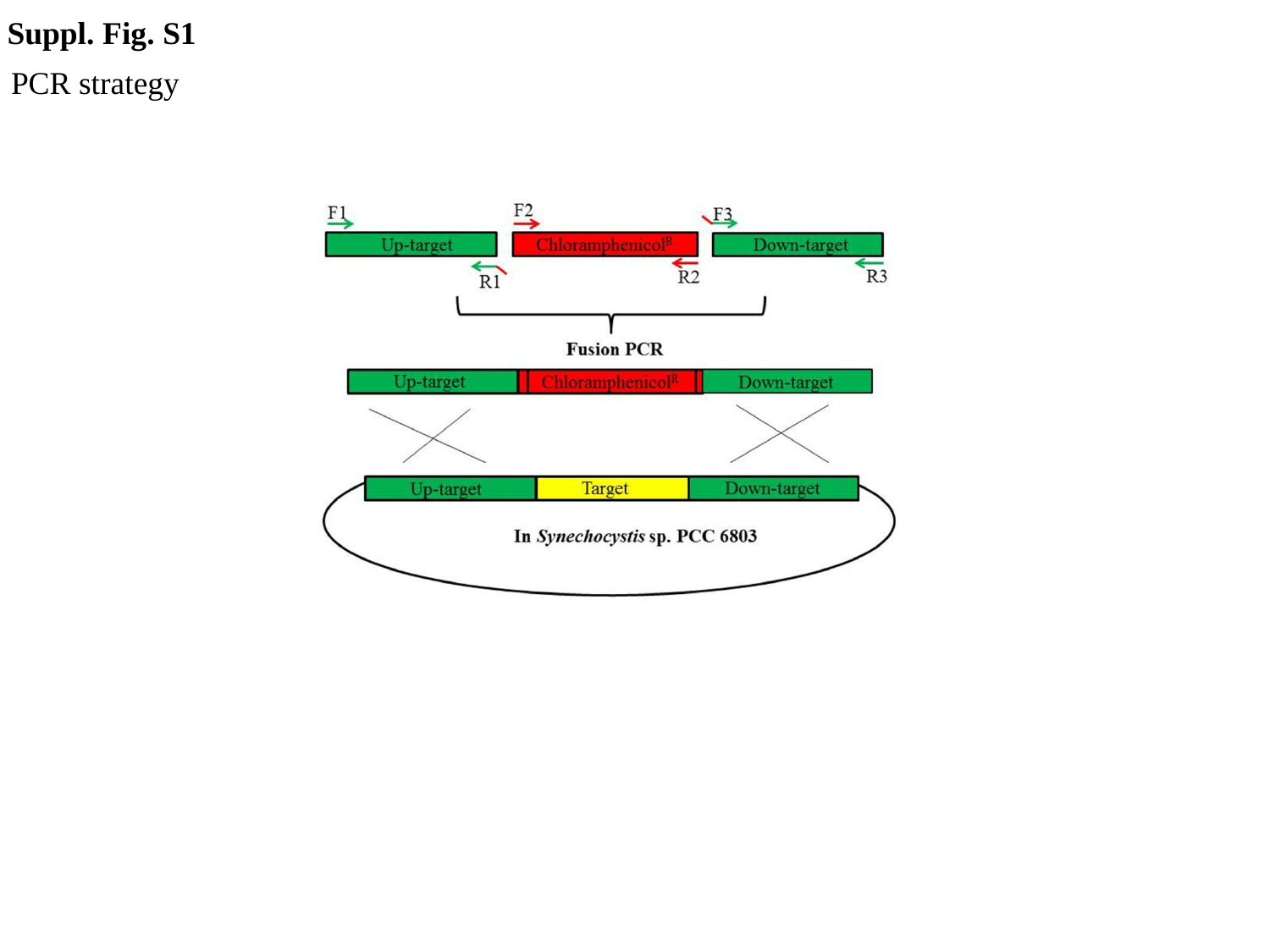

Suppl. Fig. S1
PCR strategy

## Slide 2
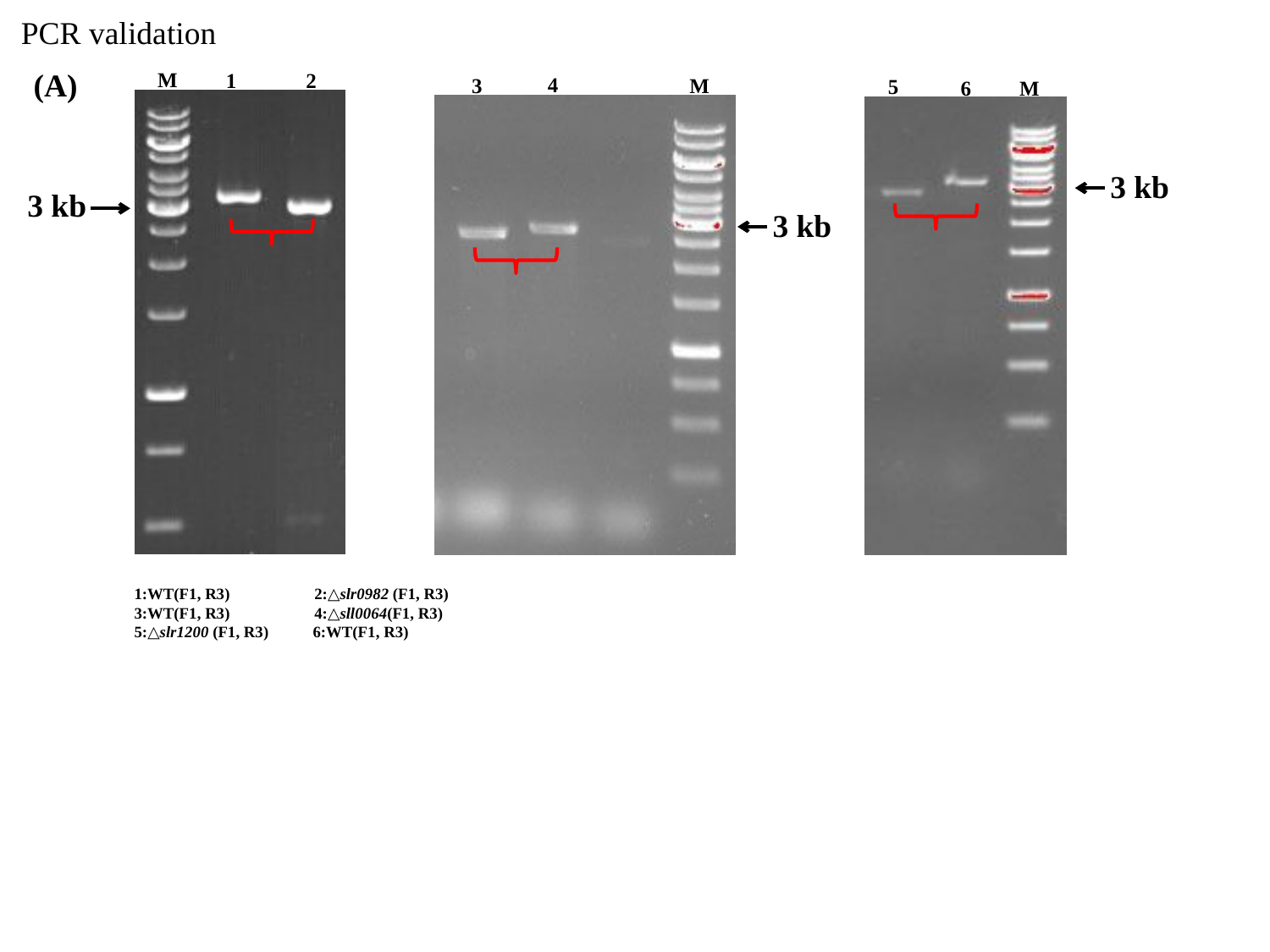

PCR validation
(A)
M
1
2
3 kb
4
M
3
5
6
M
3 kb
3 kb
1:WT(F1, R3) 2:△slr0982 (F1, R3)
3:WT(F1, R3) 4:△sll0064(F1, R3)
5:△slr1200 (F1, R3) 6:WT(F1, R3)

## Slide 3
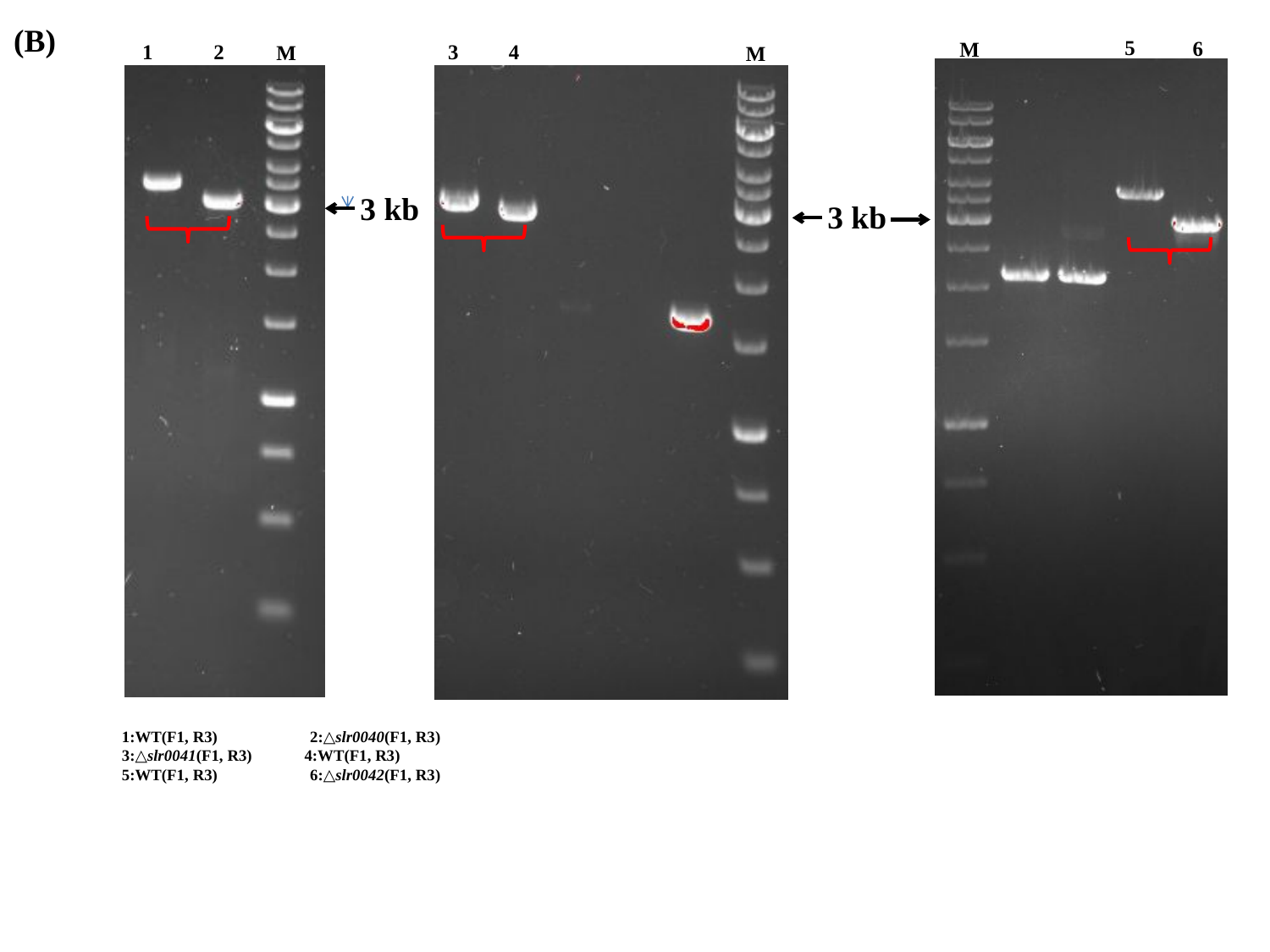

(B)
5
6
M
1
2
3
4
M
M
3 kb
3 kb
1:WT(F1, R3) 2:△slr0040(F1, R3)
3:△slr0041(F1, R3) 4:WT(F1, R3)
5:WT(F1, R3) 6:△slr0042(F1, R3)

## Slide 4
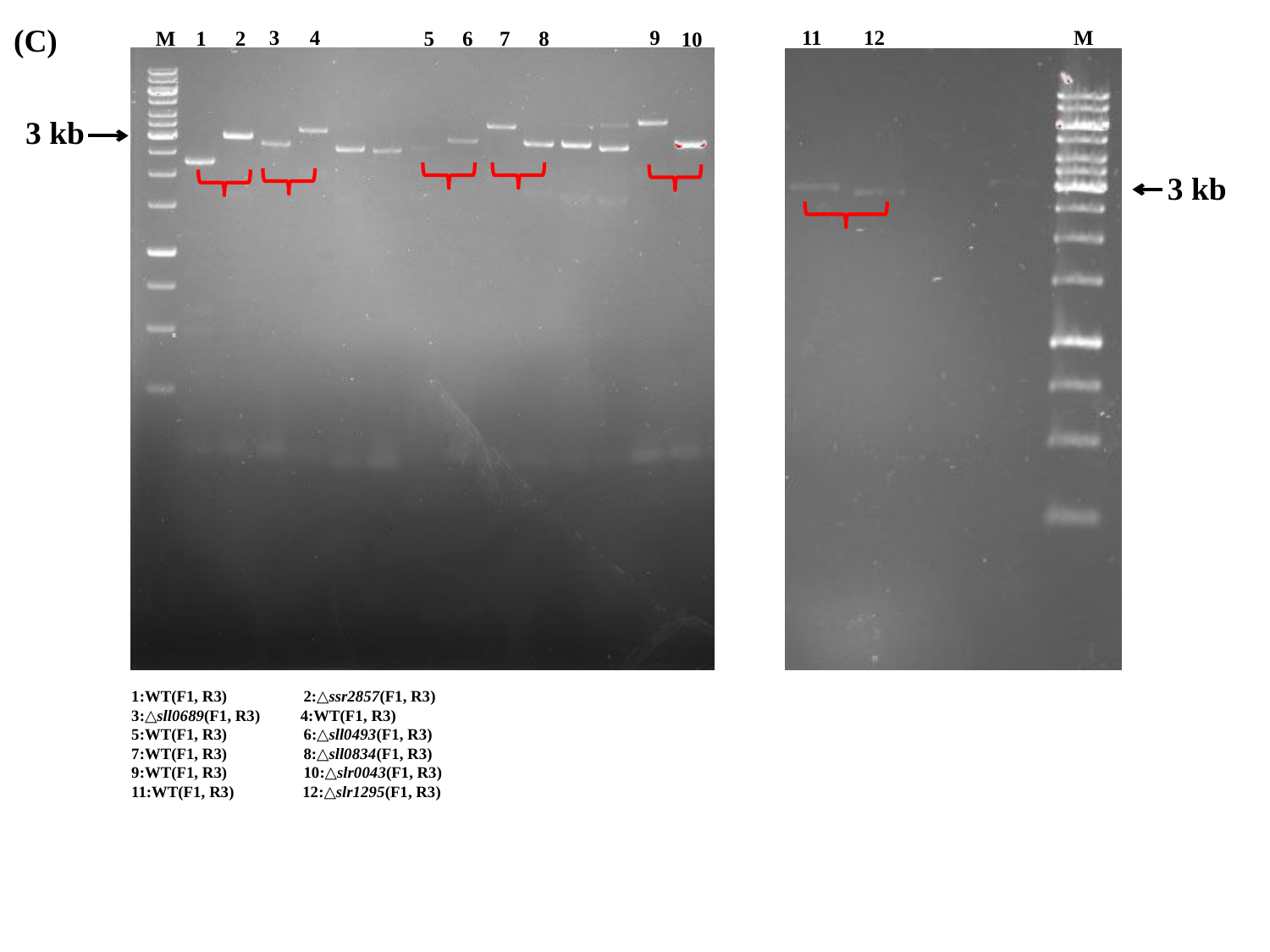

10
(C)
12
 3
4
M
9
11
 5
6
 7
 8
M
1
 2
3 kb
3 kb
1:WT(F1, R3) 2:△ssr2857(F1, R3)
3:△sll0689(F1, R3) 4:WT(F1, R3)
5:WT(F1, R3) 6:△sll0493(F1, R3)
7:WT(F1, R3) 8:△sll0834(F1, R3)
9:WT(F1, R3) 10:△slr0043(F1, R3)
11:WT(F1, R3) 12:△slr1295(F1, R3)

## Slide 5
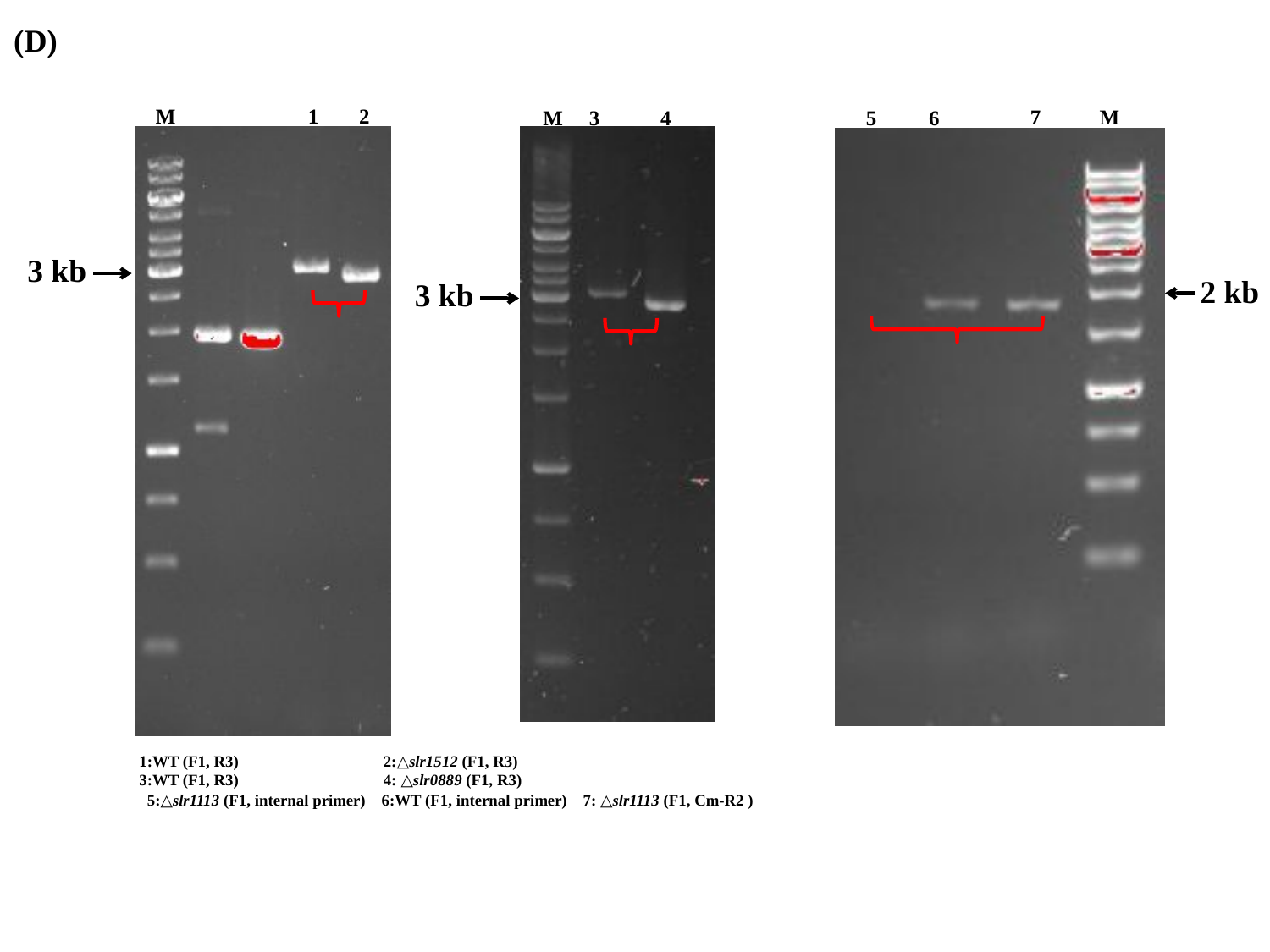

(D)
M
1
 2
M
 7
M
 3
5
 6
 4
3 kb
2 kb
3 kb
1:WT (F1, R3) 2:△slr1512 (F1, R3)
3:WT (F1, R3) 4: △slr0889 (F1, R3)
 5:△slr1113 (F1, internal primer) 6:WT (F1, internal primer) 7: △slr1113 (F1, Cm-R2 )

## Slide 6
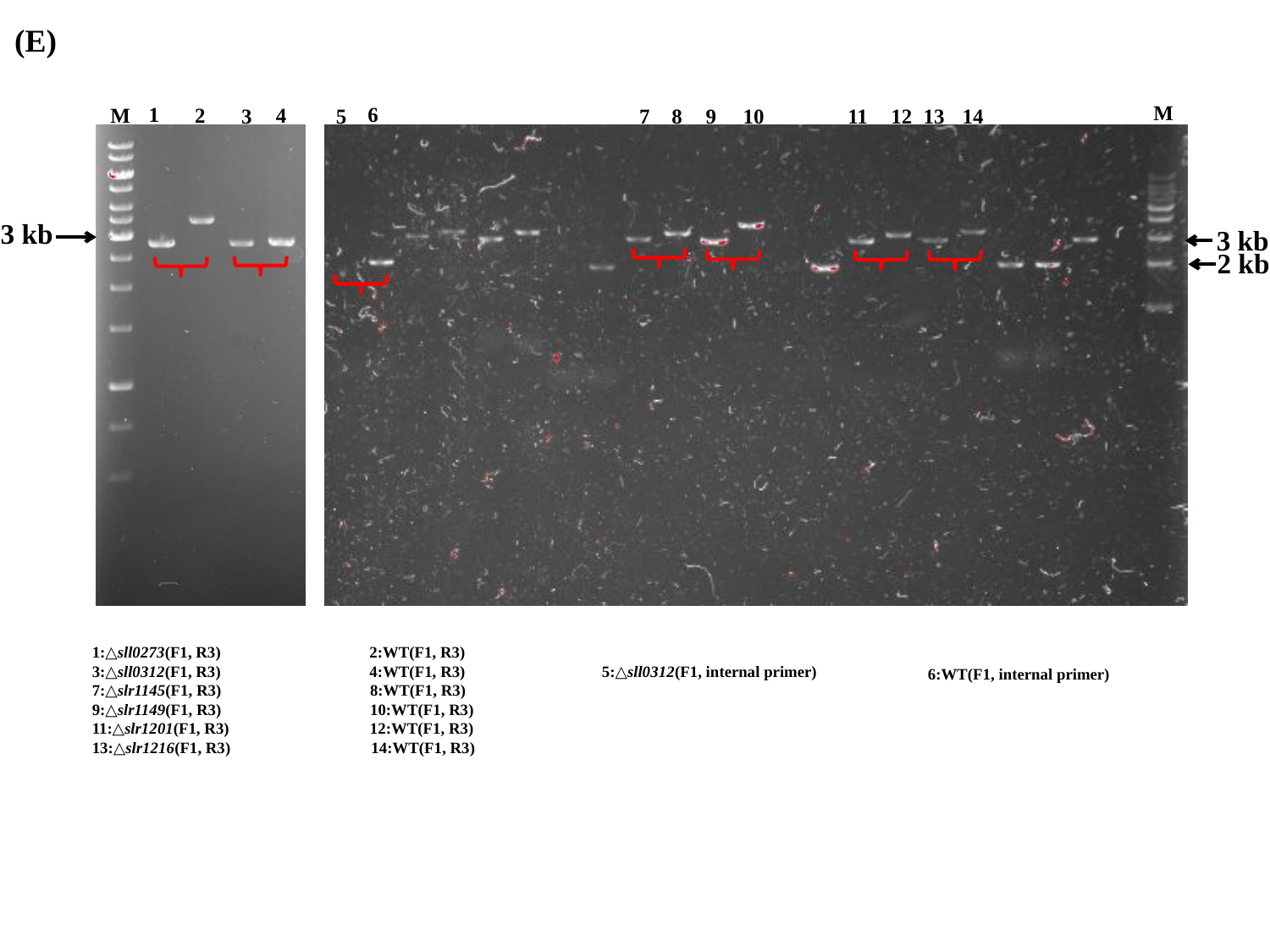

(E)
M
1
6
M
4
 2
3
5
7
8
9
10
11
12
13
14
3 kb
3 kb
2 kb
1:△sll0273(F1, R3) 2:WT(F1, R3)
3:△sll0312(F1, R3) 4:WT(F1, R3) 5:△sll0312(F1, internal primer)
7:△slr1145(F1, R3) 8:WT(F1, R3)
9:△slr1149(F1, R3) 10:WT(F1, R3)
11:△slr1201(F1, R3) 12:WT(F1, R3)
13:△slr1216(F1, R3) 14:WT(F1, R3)
6:WT(F1, internal primer)

## Slide 7
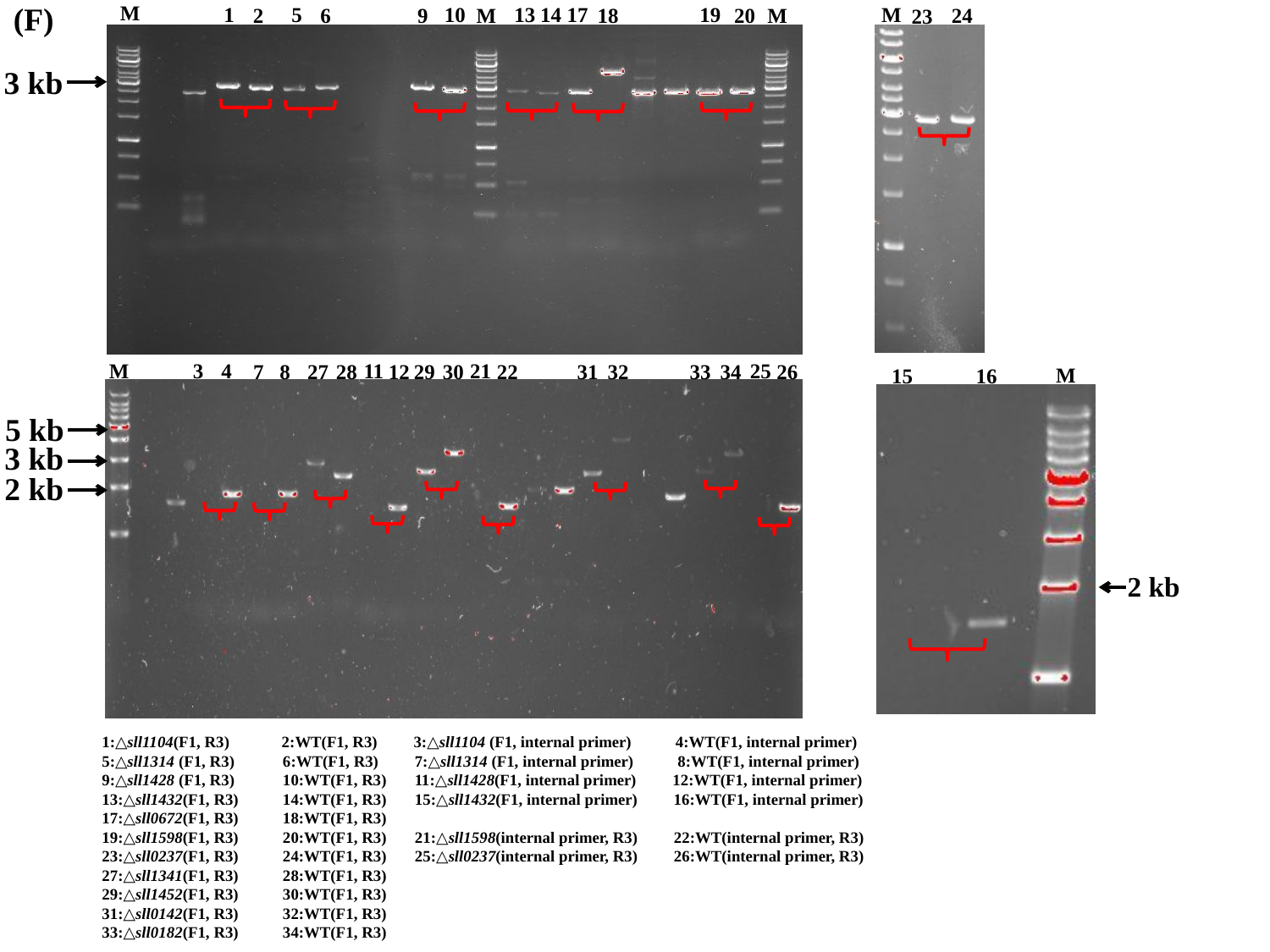

(F)
M
1
5
10
13
14
19
17
M
6
9
18
M
2
20
24
M
23
3 kb
M
21
3
11
25
4
27
29
30
31
32
33
34
26
7 8
22
28
12
M
16
15
5 kb
3 kb
2 kb
2 kb
1:△sll1104(F1, R3) 2:WT(F1, R3) 3:△sll1104 (F1, internal primer) 4:WT(F1, internal primer)
5:△sll1314 (F1, R3) 6:WT(F1, R3) 7:△sll1314 (F1, internal primer) 8:WT(F1, internal primer)
9:△sll1428 (F1, R3) 10:WT(F1, R3) 11:△sll1428(F1, internal primer) 12:WT(F1, internal primer)
13:△sll1432(F1, R3) 14:WT(F1, R3) 15:△sll1432(F1, internal primer) 16:WT(F1, internal primer)
17:△sll0672(F1, R3) 18:WT(F1, R3)
19:△sll1598(F1, R3) 20:WT(F1, R3) 21:△sll1598(internal primer, R3) 22:WT(internal primer, R3)
23:△sll0237(F1, R3) 24:WT(F1, R3) 25:△sll0237(internal primer, R3) 26:WT(internal primer, R3)
27:△sll1341(F1, R3) 28:WT(F1, R3)
29:△sll1452(F1, R3) 30:WT(F1, R3)
31:△sll0142(F1, R3) 32:WT(F1, R3)
33:△sll0182(F1, R3) 34:WT(F1, R3)

## Slide 8
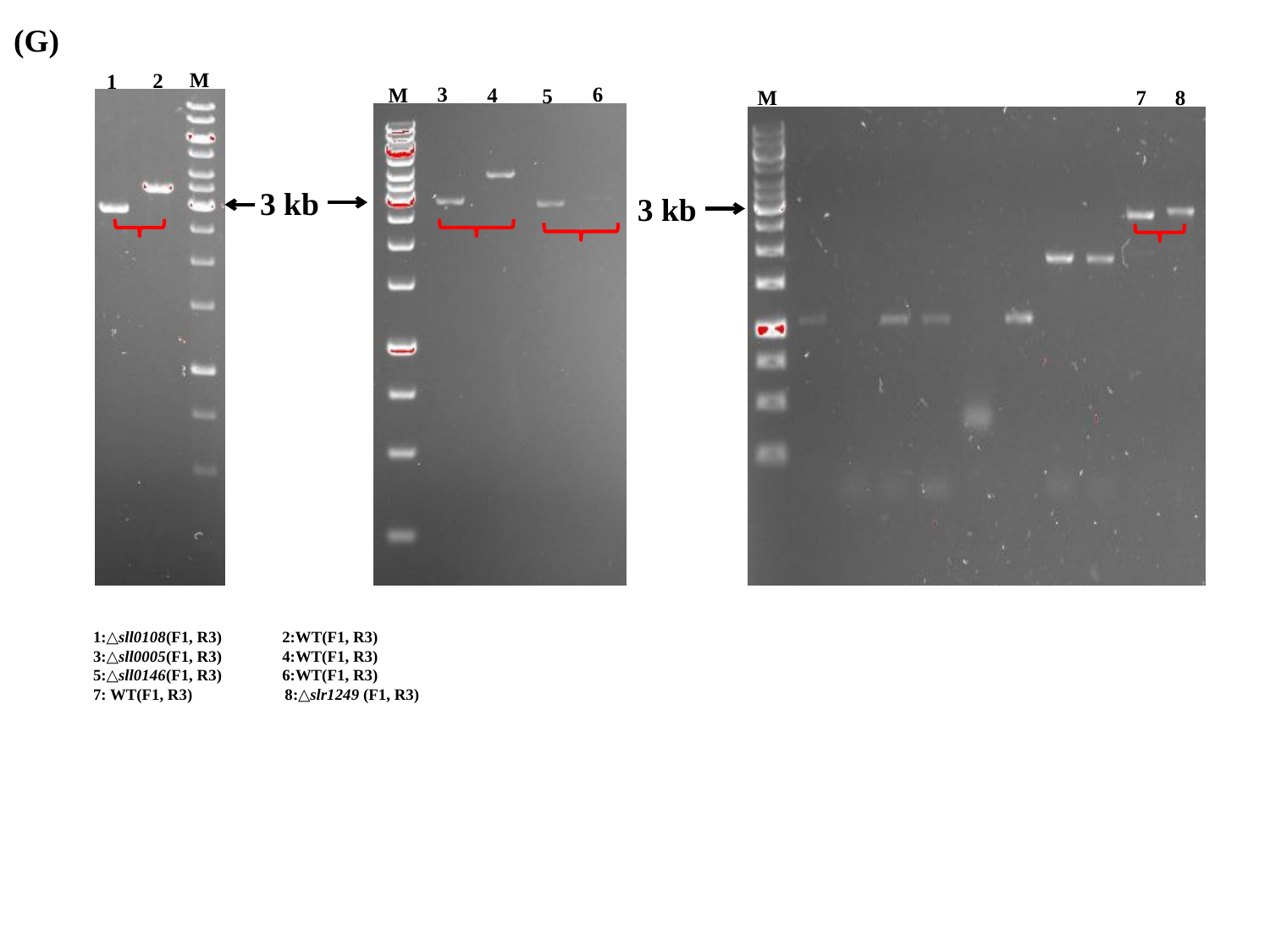

(G)
M
2
1
3
6
M
4
5
7
M
8
3 kb
3 kb
1:△sll0108(F1, R3) 2:WT(F1, R3)
3:△sll0005(F1, R3) 4:WT(F1, R3)
5:△sll0146(F1, R3) 6:WT(F1, R3)
7: WT(F1, R3) 8:△slr1249 (F1, R3)

## Slide 9
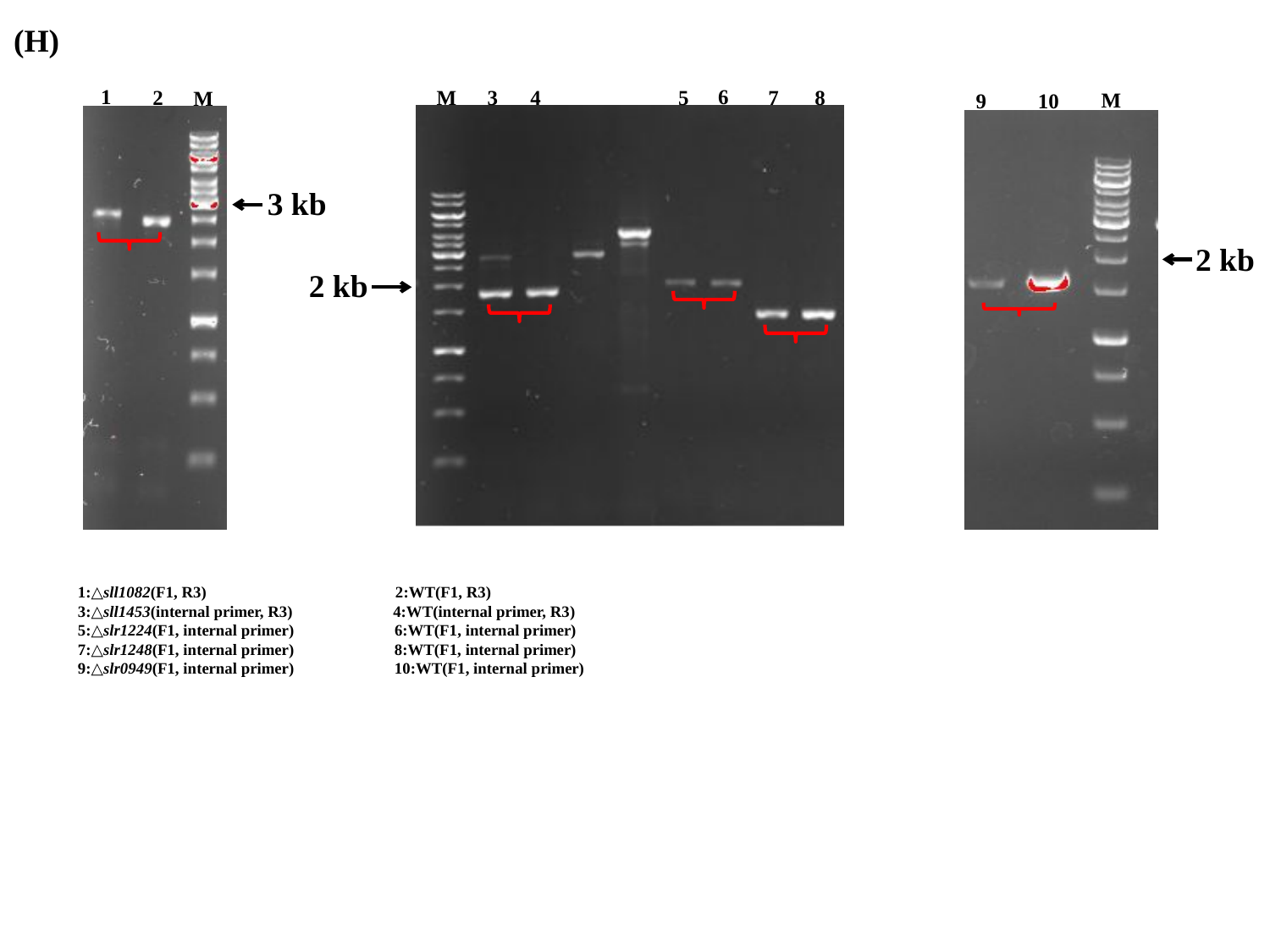

(H)
1
6
2
M
3
4
5
7
8
M
M
9
10
3 kb
2 kb
2 kb
1:△sll1082(F1, R3) 2:WT(F1, R3)
3:△sll1453(internal primer, R3) 4:WT(internal primer, R3)
5:△slr1224(F1, internal primer) 6:WT(F1, internal primer)
7:△slr1248(F1, internal primer) 8:WT(F1, internal primer)
9:△slr0949(F1, internal primer) 10:WT(F1, internal primer)

## Slide 10
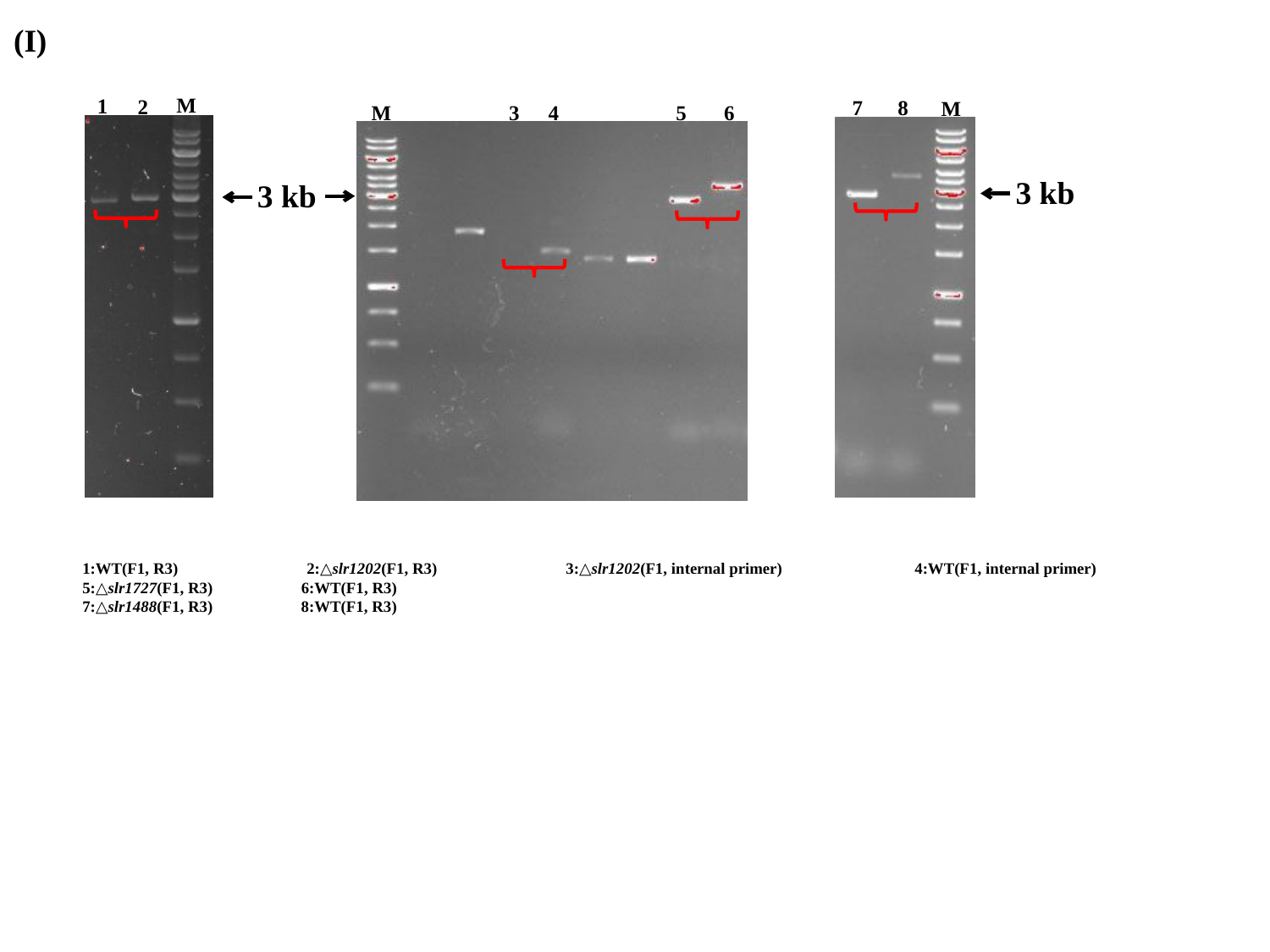

(I)
M
1
2
7
8
M
M
3
4
5
6
3 kb
3 kb
1:WT(F1, R3) 2:△slr1202(F1, R3) 3:△slr1202(F1, internal primer) 4:WT(F1, internal primer)
5:△slr1727(F1, R3) 6:WT(F1, R3)
7:△slr1488(F1, R3) 8:WT(F1, R3)

## Slide 11
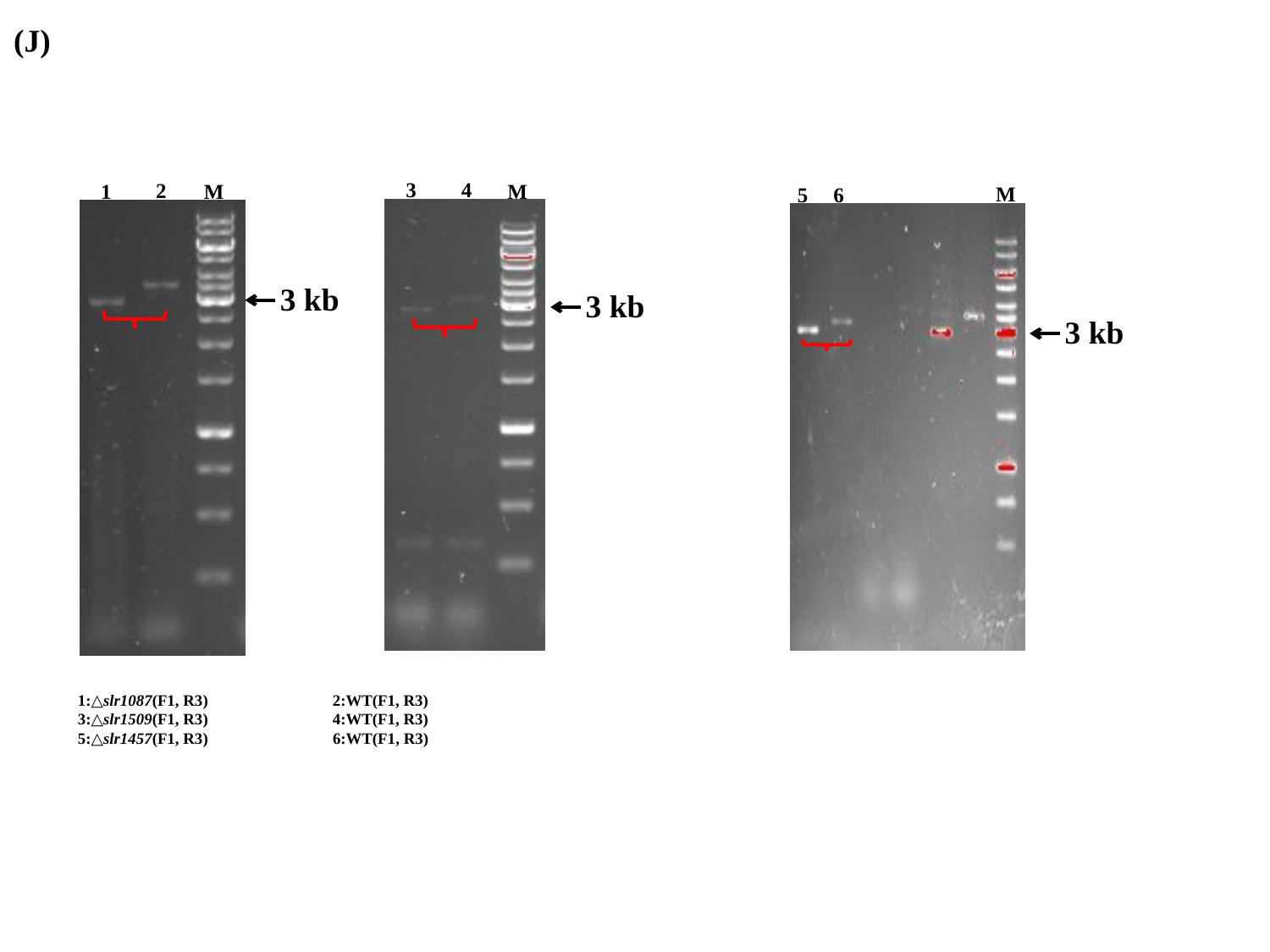

(J)
3
4
2
M
M
1
M
5
6
3 kb
3 kb
3 kb
1:△slr1087(F1, R3) 2:WT(F1, R3)
3:△slr1509(F1, R3) 4:WT(F1, R3)
5:△slr1457(F1, R3) 6:WT(F1, R3)

## Slide 12
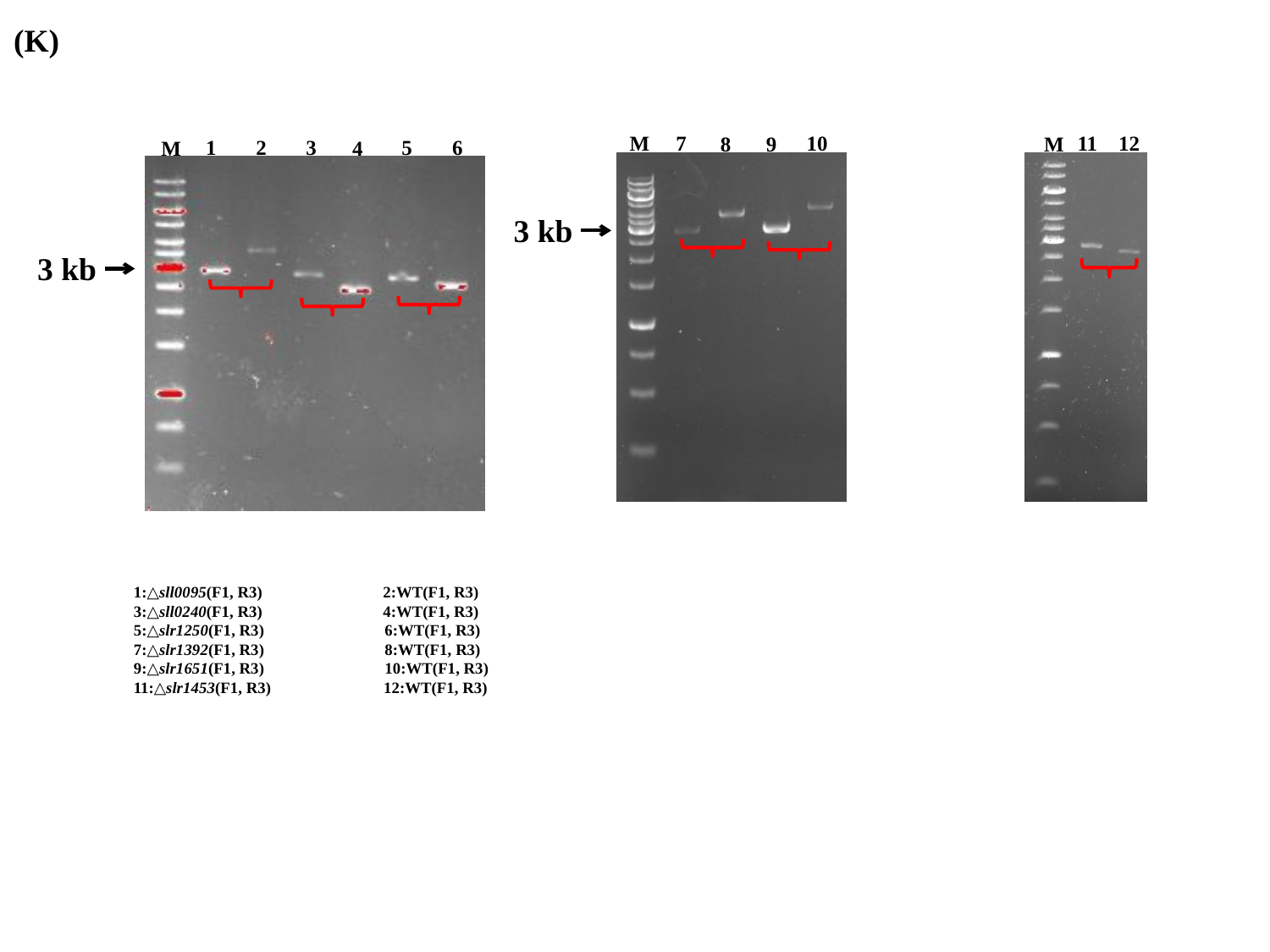

(K)
M
7
11
12
10
8
M
9
1
2
3
5
6
4
M
3 kb
3 kb
1:△sll0095(F1, R3) 2:WT(F1, R3)
3:△sll0240(F1, R3) 4:WT(F1, R3)
5:△slr1250(F1, R3) 6:WT(F1, R3)
7:△slr1392(F1, R3) 8:WT(F1, R3)
9:△slr1651(F1, R3) 10:WT(F1, R3)
11:△slr1453(F1, R3) 12:WT(F1, R3)

## Slide 13
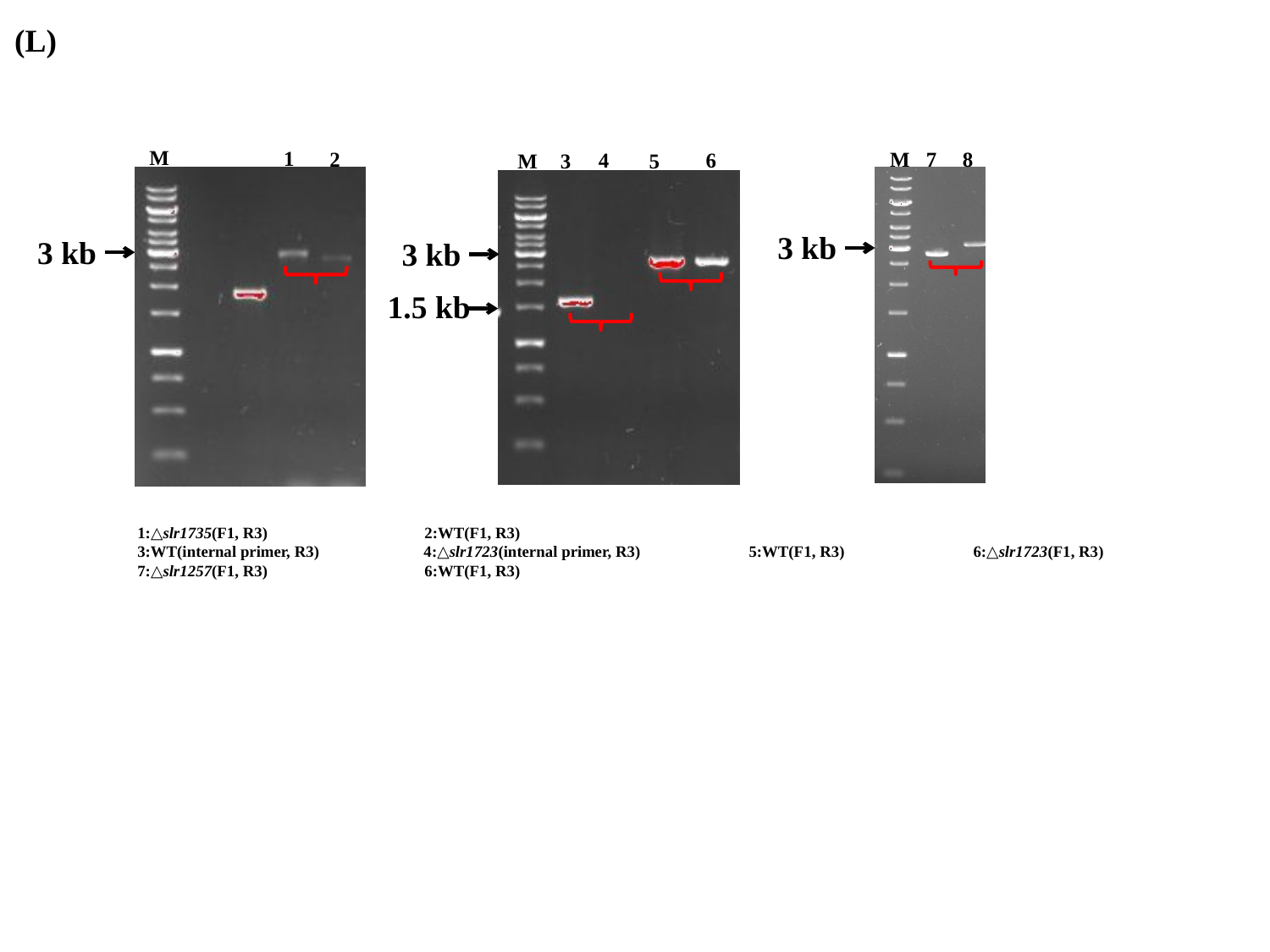

(L)
M
1
2
M
7
8
4
6
M
3
5
3 kb
3 kb
3 kb
1.5 kb
1:△slr1735(F1, R3) 2:WT(F1, R3)
3:WT(internal primer, R3) 4:△slr1723(internal primer, R3) 5:WT(F1, R3) 6:△slr1723(F1, R3)
7:△slr1257(F1, R3) 6:WT(F1, R3)

## Slide 14
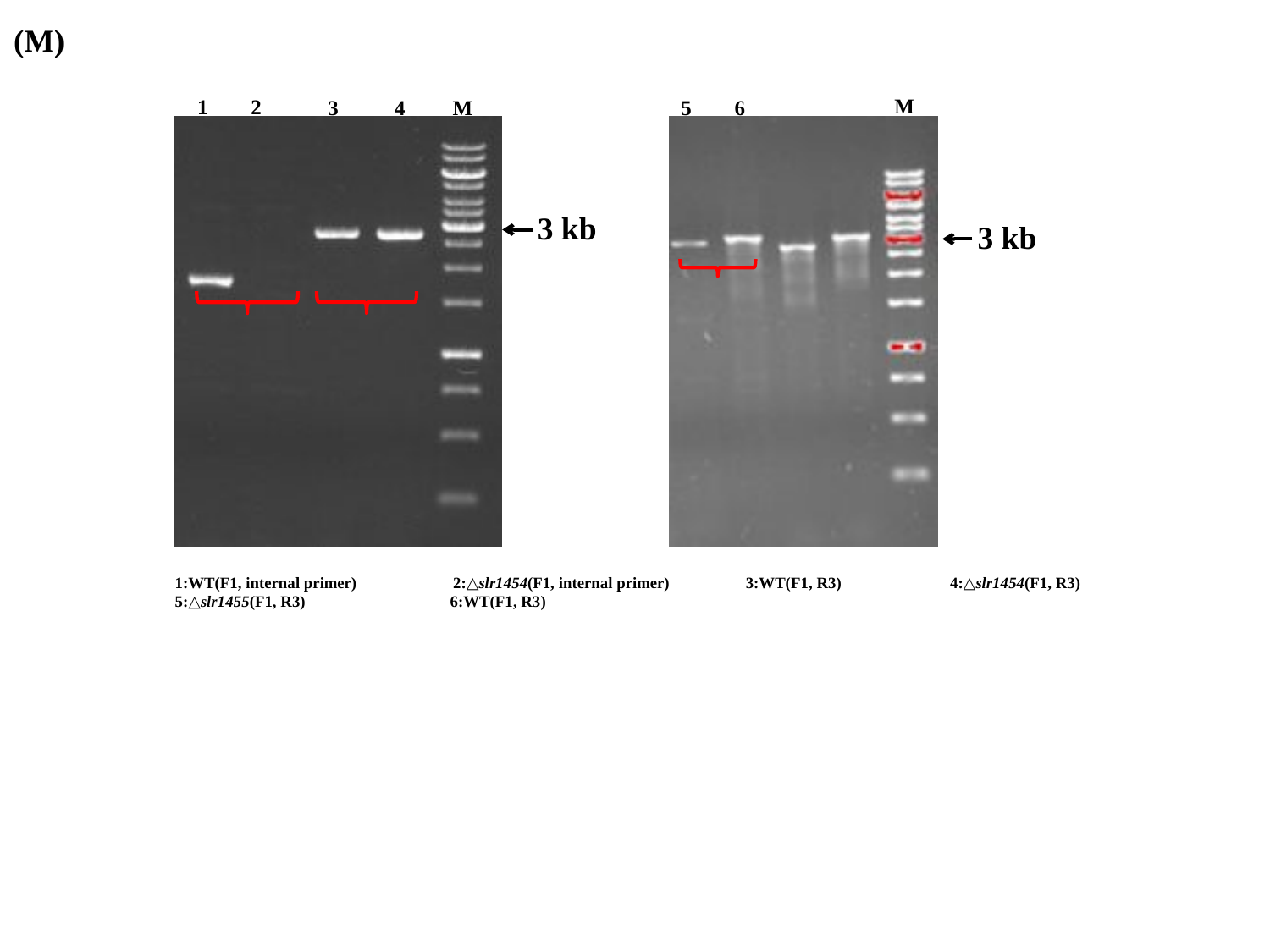

(M)
M
1
2
3
4
6
5
M
3 kb
3 kb
1:WT(F1, internal primer) 2:△slr1454(F1, internal primer) 3:WT(F1, R3) 4:△slr1454(F1, R3)
5:△slr1455(F1, R3) 6:WT(F1, R3)

## Slide 15
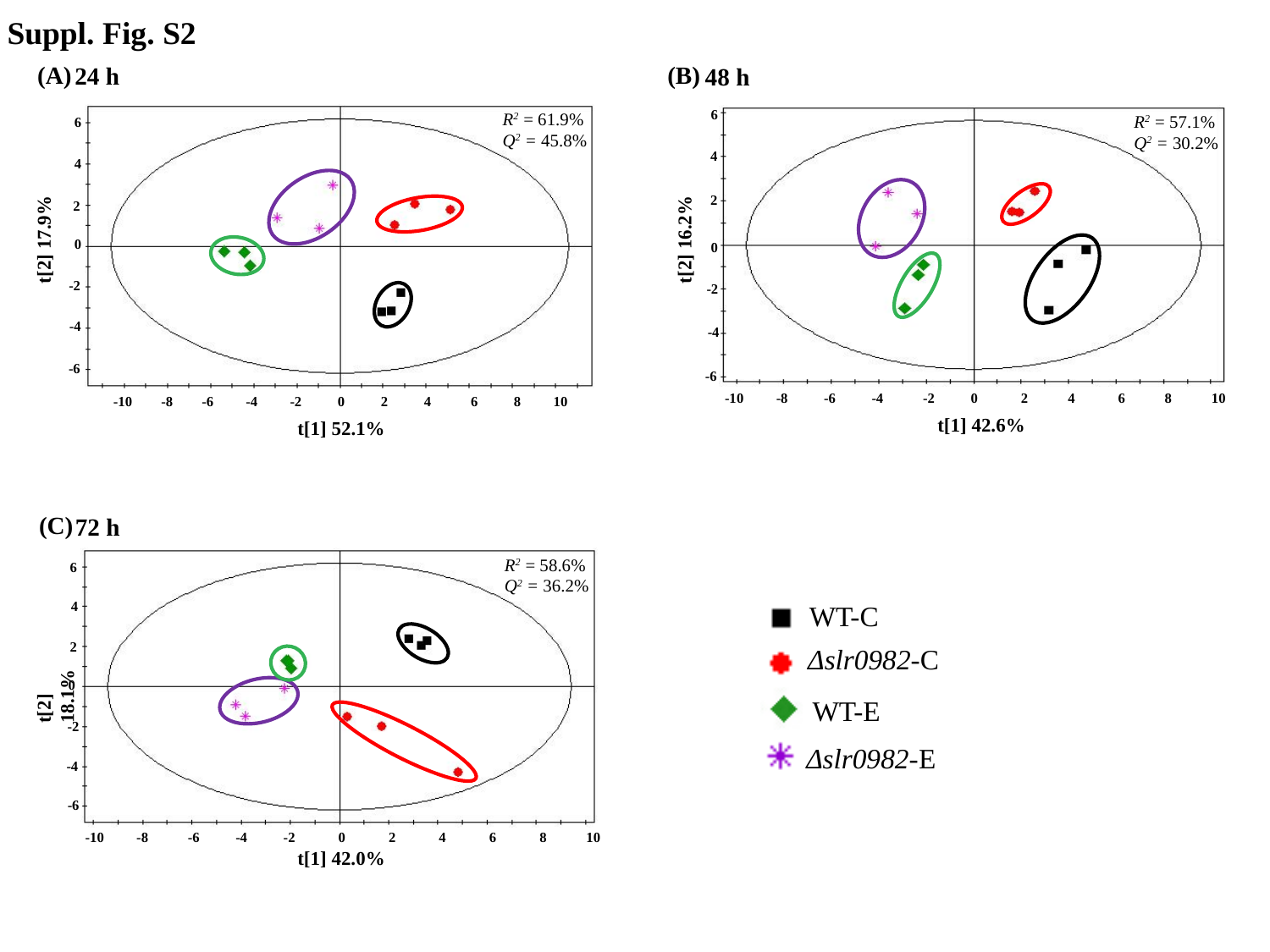

Suppl. Fig. S2
(A)
(B)
6
4
t[2] 16.2%
2
0
-2
-4
-6
 -10 -8 -6 -4 -2 0 2 4 6 8 10
t[1] 42.6%
24 h
48 h
R2 = 61.9%
Q2 = 45.8%
R2 = 57.1%
Q2 = 30.2%
6
4
t[2] 17.9%
2
0
-2
-4
 -10 -8 -6 -4 -2 0 2 4 6 8 10
t[1] 52.1%
-6
(C)
72 h
R2 = 58.6%
Q2 = 36.2%
6
4
2
0
-2
-4
-6
 -10 -8 -6 -4 -2 0 2 4 6 8 10
t[1] 42.0%
WT-C
Δslr0982-C
WT-E
Δslr0982-E
t[2] 18.1%

## Slide 16
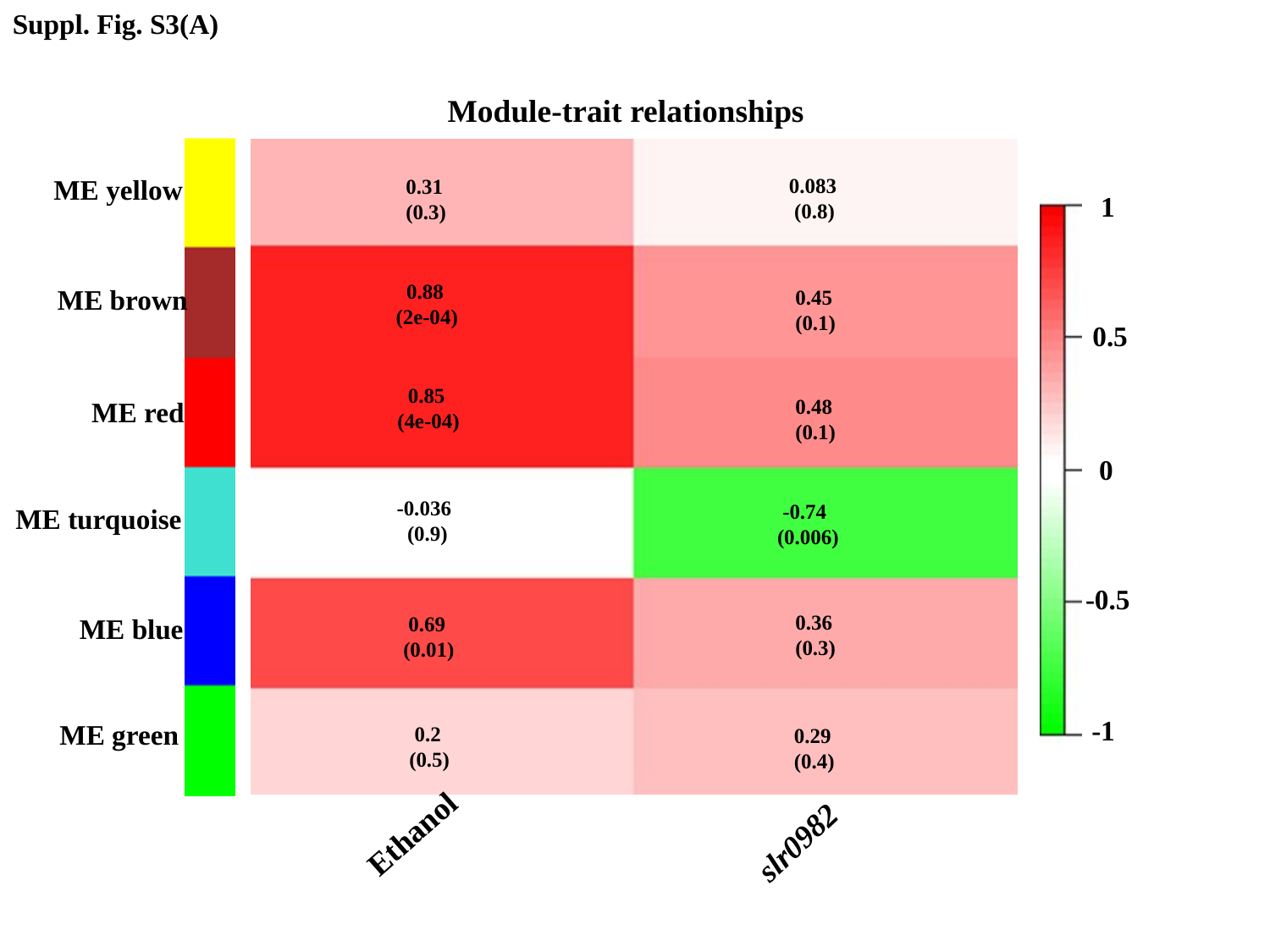

Suppl. Fig. S3(A)
Module-trait relationships
0.083
 (0.8)
ME yellow
0.31
(0.3)
1
 0.88
 (2e-04)
ME brown
 0.45
 (0.1)
0.5
 0.85
 (4e-04)
 0.48
 (0.1)
ME red
0
 -0.036
 (0.9)
 -0.74
 (0.006)
ME turquoise
-0.5
 0.36
 (0.3)
 0.69
 (0.01)
ME blue
-1
ME green
 0.2
 (0.5)
 0.29
 (0.4)
Ethanol
slr0982

## Slide 17
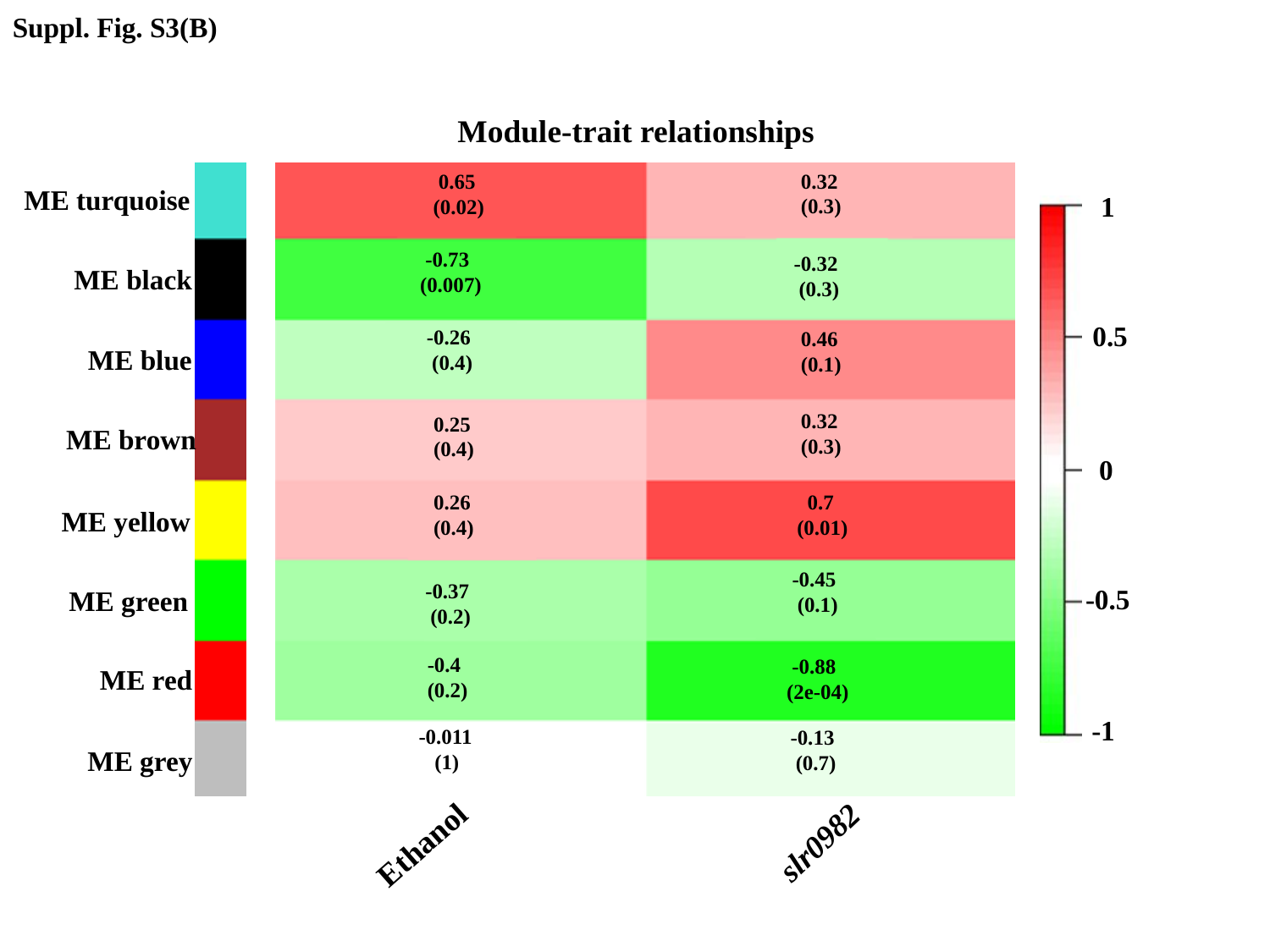

Suppl. Fig. S3(B)
Module-trait relationships
 0.32
 (0.3)
 0.65
 (0.02)
 -0.73
 (0.007)
 -0.32
 (0.3)
 -0.26
 (0.4)
 0.46
 (0.1)
 0.32
 (0.3)
 0.25
 (0.4)
 0.26
 (0.4)
 0.7
 (0.01)
 -0.45
 (0.1)
 -0.37
 (0.2)
 -0.4
 (0.2)
 -0.88
 (2e-04)
 -0.011
 (1)
 -0.13
 (0.7)
ME turquoise
1
ME black
0.5
ME blue
ME brown
0
ME yellow
-0.5
ME green
ME red
-1
ME grey
Ethanol
slr0982

## Slide 18
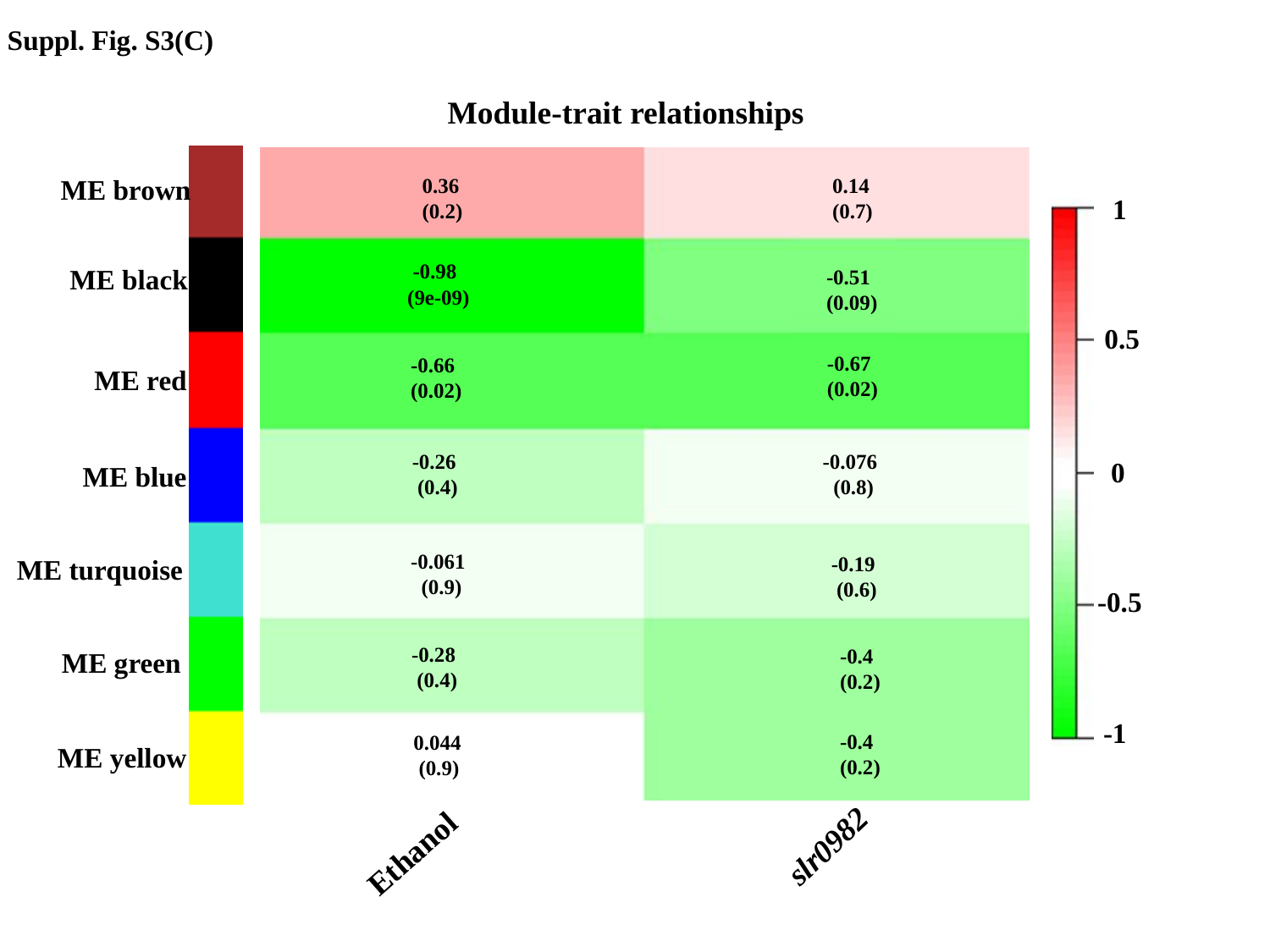

Suppl. Fig. S3(C)
Module-trait relationships
0.36
(0.2)
ME brown
0.14
(0.7)
1
 -0.98
(9e-09)
ME black
 -0.51
 (0.09)
0.5
 -0.67
 (0.02)
 -0.66
 (0.02)
ME red
 -0.26
 (0.4)
 -0.076
 (0.8)
0
ME blue
 -0.061
 (0.9)
 -0.19
 (0.6)
ME turquoise
-0.5
 -0.28
 (0.4)
 -0.4
 (0.2)
ME green
-1
 -0.4
 (0.2)
 0.044
 (0.9)
ME yellow
Ethanol
slr0982

## Slide 19
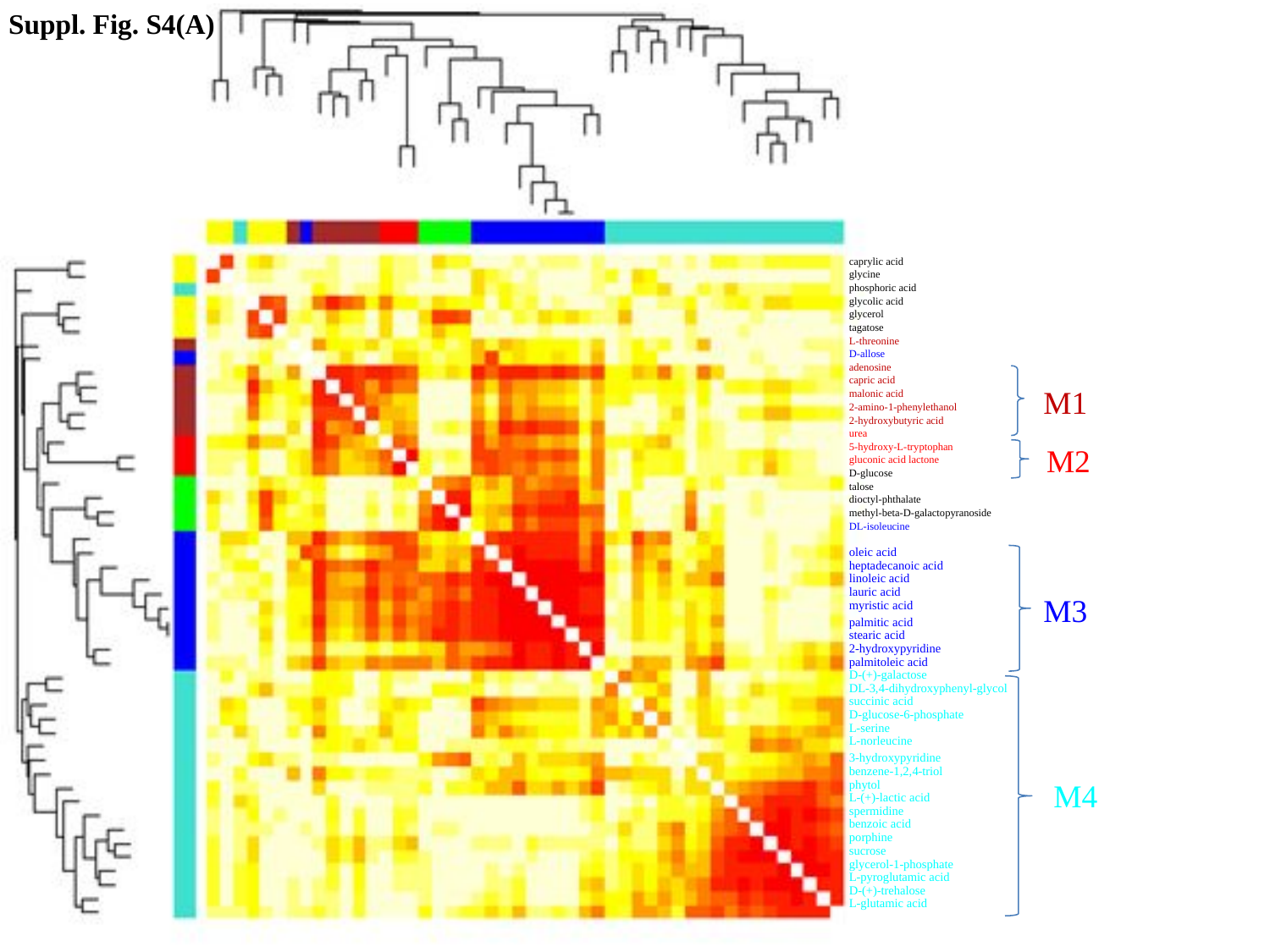

Suppl. Fig. S4(A)
caprylic acid
glycine
phosphoric acid
glycolic acid
glycerol
tagatose
L-threonine
D-allose
adenosine
capric acid
malonic acid
2-amino-1-phenylethanol
2-hydroxybutyric acid
urea
5-hydroxy-L-tryptophan
gluconic acid lactone
D-glucose
talose
dioctyl-phthalate
methyl-beta-D-galactopyranoside
DL-isoleucine
oleic acid
heptadecanoic acid
linoleic acid
lauric acid
myristic acid
palmitic acid
stearic acid
2-hydroxypyridine
palmitoleic acid
D-(+)-galactose
DL-3,4-dihydroxyphenyl-glycol
succinic acid
D-glucose-6-phosphate
L-serine
L-norleucine
3-hydroxypyridine
benzene-1,2,4-triol
phytol
L-(+)-lactic acid
spermidine
benzoic acid
porphine
sucrose
glycerol-1-phosphate
L-pyroglutamic acid
D-(+)-trehalose
L-glutamic acid
 M1
 M2
 M3
 M4

## Slide 20
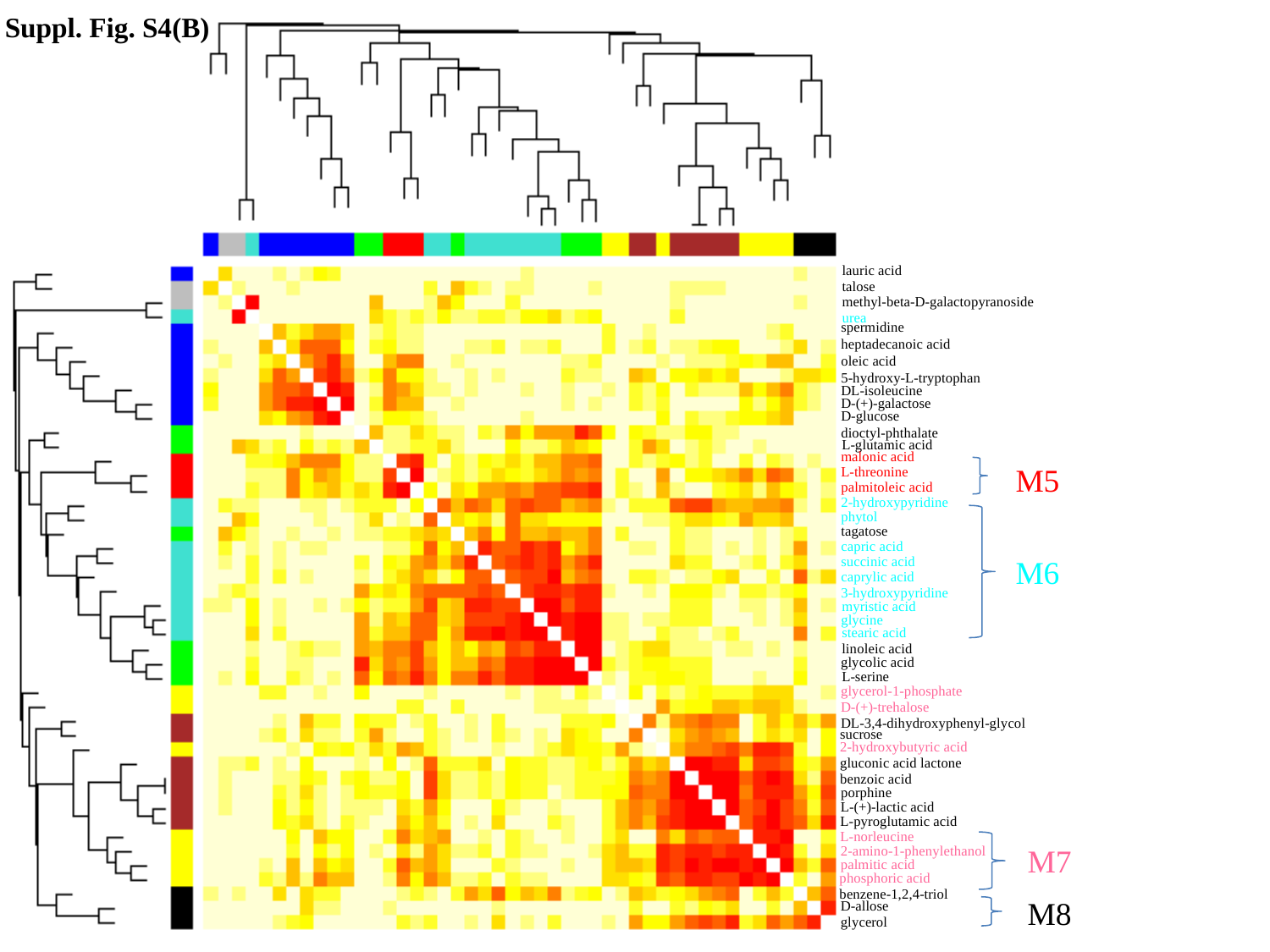

Suppl. Fig. S4(B)
lauric acid
talose
methyl-beta-D-galactopyranoside
urea
spermidine
heptadecanoic acid
oleic acid
5-hydroxy-L-tryptophan
DL-isoleucine
D-(+)-galactose
D-glucose
dioctyl-phthalate
L-glutamic acid
malonic acid
L-threonine
palmitoleic acid
2-hydroxypyridine
phytol
tagatose
capric acid
succinic acid
caprylic acid
3-hydroxypyridine
myristic acid
glycine
stearic acid
linoleic acid
glycolic acid
L-serine
glycerol-1-phosphate
D-(+)-trehalose
DL-3,4-dihydroxyphenyl-glycol
sucrose
2-hydroxybutyric acid
gluconic acid lactone
benzoic acid
porphine
L-(+)-lactic acid
L-pyroglutamic acid
L-norleucine
2-amino-1-phenylethanol
palmitic acid
phosphoric acid
benzene-1,2,4-triol
D-allose
glycerol
M5
M6
M7
M8

## Slide 21
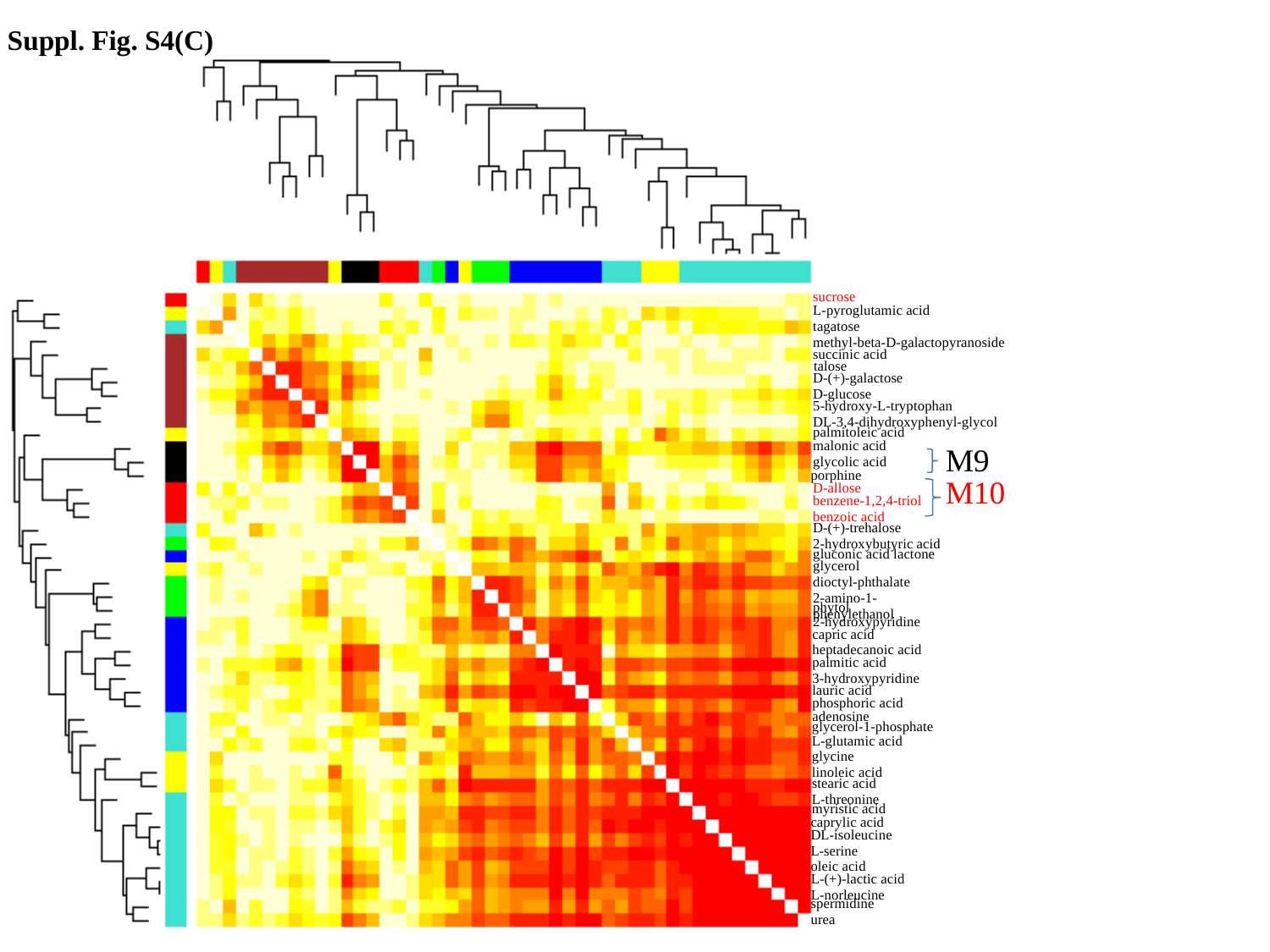

Suppl. Fig. S4(C)
sucrose
L-pyroglutamic acid
tagatose
methyl-beta-D-galactopyranoside
succinic acid
D-(+)-galactose
D-glucose
5-hydroxy-L-tryptophan
DL-3,4-dihydroxyphenyl-glycol
palmitoleic acid
malonic acid
glycolic acid
porphine
D-allose
benzene-1,2,4-triol
benzoic acid
D-(+)-trehalose
2-hydroxybutyric acid
gluconic acid lactone
glycerol
dioctyl-phthalate
2-amino-1-phenylethanol
phytol
2-hydroxypyridine
capric acid
heptadecanoic acid
palmitic acid
3-hydroxypyridine
lauric acid
phosphoric acid
adenosine
glycerol-1-phosphate
L-glutamic acid
glycine
linoleic acid
stearic acid
L-threonine
myristic acid
caprylic acid
DL-isoleucine
L-serine
oleic acid
L-(+)-lactic acid
L-norleucine
spermidine
urea
talose
M9
M10
